# Ferroptosis-Mediated Radiosensitization in Glioblastoma: Preclinical Insights and Translational Opportunities

**DOI:** 10.3390/ijms27219505

**Published:** 2026-10-24

**Authors:** Olivia E. Gilbert, Henry F. Fowler, Asish Madarapu, Max E. Reed, Ali D. Fahim, Sebastian John, Colette J. Shen, Dominique M. O. Higgins

**Affiliations:** 1 Department of Neurosurgery, University of North Carolina, Chapel Hill, NC 27599, USA; 2 Department of Neurosurgery, Weill Cornell Medicine, New York, NY 10065, USA; 3 Department of Radiation Oncology, University of North Carolina, Chapel Hill, NC 27599, USA

**Keywords:** ferroptosis, glioblastoma, radiosensitization, radiotherapy, nanoparticle, biomarkers, FLASH

## Abstract

Glioblastoma (GBM) remains the most common and most aggressive primary malignant brain tumor in adults, and despite maximal safe resection with adjuvant radiotherapy (RT) and temozolomide (TMZ), median overall survival remains 12–15 months with near-universal recurrence. Ferroptosis, an iron-dependent form of regulated cell death driven by the accumulation of lipid peroxides, has emerged as a distinctive vulnerability in GBM, whose iron-avid phenotype, polyunsaturated fatty acid-rich milieu, and high oxygen consumption create a permissive substrate for lipid peroxidation. In this review, we synthesize preclinical evidence positioning ferroptosis as a determinant of GBM radiosensitivity and evaluate strategies to exploit it. We first outline core ferroptosis biology and GBM-specific modifiers of ferroptotic susceptibility, including TFR1-mediated iron import, grade-dependent glutathione peroxidase 4 (GPX4) expression, acyl-coenzyme A synthetase long-chain family member 4 (ACSL4) levels, transsulfuration, NRF2, p53, non-coding RNAs, and the relative resistance of mesenchymal tumors and glioma stem cells. We then examine mechanisms of radiation-induced ferroptosis, in which RT upregulates ACSL4 and depletes glutathione (GSH) while simultaneously provoking adaptive responses that induce the cystine transporter solute carrier family 7 member 11 (SLC7A11) and GPX4, alongside hypoxia-, IFI16/HMOX1-, and lncRNA-mediated programs that sustain radioresistance, noting that recurrent tumors display a ferroptosis-primed profile after chemoradiation. Radiosensitization strategies are reviewed across pharmacologic ferroptosis inducers (classes I-IV), repurposed agents, NRF2 inhibition, genetic targeting of ferroptosis mediators, and blood–brain barrier-penetrant nanoplatforms that couple iron delivery, high-Z radiation deposition, and antioxidant disruption. We further address the immunologic consequences of ferroptosis within the immunosuppressive GBM microenvironment, the countervailing problem of radiation-induced brain injury and candidate neuroprotective approaches, the potential convergence of FLASH RT with iron-dependent differential sparing, and comparative evidence of ferroptosis-RT synergy in other treatment-resistant malignancies. Finally, we appraise candidate biomarkers and ferroptosis-related gene signatures, and close by addressing open questions and current translational hurdles facing the field.

## 1. Introduction

Glioblastoma (GBM) is the most common and most aggressive malignant primary brain tumor in adults, accounting for 58.6% of all malignant central nervous system (CNS) tumors [1,2]. According to the fifth edition of the *WHO Classification of Tumors of the Central Nervous System* (CNS), published in 2021, GBM isocitrate dehydrogenase-wildtype (IDH-wt) is diagnosed when diffuse astrocytic glioma tumor tissue is IDH-wt and there is concurrent microvascular proliferation, necrosis, TERT promoter mutation, EGFR gene amplification, or +7/−10 chromosomal changes [3]. These changes in diagnostic criteria reflect the recent increase in the importance of genetic and molecular parameters in the identification and management of GBM. The current standard of care for patients with GBM involves maximal safe resection with 60 Gy of adjuvant radiotherapy (RT) over 6 weeks and 75 mg/m^2^/day of temozolomide (TMZ) for 6 weeks, followed by maintenance TMZ for six 28-day cycles of 150–200 mg/m^2^/day for 5 days [4]. GBM patients with IDH mutations historically exhibit a better response to TMZ and improved prognosis relative to IDH-wildtype patients [5]. Despite aggressive therapy, median overall survival (OS) is 12–15 months and recurrence is near-universal [6]. Resistance to RT and chemotherapy is driven by multiple factors, including clonal evolution, DNA repair, glioblastoma stem-like cells (GSCs), metabolic gradients, spatiotemporal disorganization, hypoxia, and immunosuppressive microenvironments within heterogeneous tumor tissue [7]. Current research focuses on utilizing novel therapeutic strategies such as immunotherapy, targeted drug delivery, targeting tumor metabolism, and regulatory cell death (RCD) sensitization.

Ferroptosis, an iron-dependent form of regulated cell death characterized by lipid peroxidation, is a newly recognized vulnerability in GBM [8]. The brain’s high iron content, rich polyunsaturated fatty acid (PUFA) composition, and high oxygen consumption make it uniquely susceptible to ferroptotic cell death [8,9]. GBM is no longer regarded as a tumor of differentiated glia: current evidence favors an origin in astrocyte-like neural stem or progenitor cells of the subventricular zone and in glioma stem-like cells [10]. These tumor cells demonstrate metabolic plasticity, adapting to glucose limitation by upregulating lipid metabolism. Within the tumor microenvironment, immunosuppressive macrophages, regulatory T cells, and resistant glioma cells have demonstrated similar metabolic profiles involving oxidative stress, altered iron use, and reliance on lipid catabolism. In this setting, ferroptosis may enhance RCD of immune and glial cells [11]. In this review, we will focus on ferroptosis as a determinant of GBM sensitivity to RT and evaluate strategies to exploit this vulnerability.

## 2. Ferroptosis Brief Overview

Ferroptosis is a form of RCD characterized by the accumulation of lipid peroxides [12]. As the name implies, ferroptosis is distinguished by its dependence on iron. In the cell, ferrous iron (Fe^2+^) can participate in redox Fenton reactions, reacting with hydrogen peroxide to generate reactive oxygen species (ROS). When these ROS interact with the phospholipid-rich cell membrane, they initiate a lipid peroxidation chain reaction. Once the cell’s capacity for antioxidant detoxification can no longer keep up with the lipid peroxidation, it experiences altered ion transport, cell swelling, and ultimately plasma membrane rupture. Consistent with this mechanism, iron chelation has been shown to suppress ferroptosis [12]. Under baseline conditions, the cell’s defenses can typically protect against this oxidative damage, with the SLC7A11-GSH-GPX4 axis serving as a central component. SLC7A11, a component of the cystine–glutamate exchanger (system Xc^−^), imports cystine into the cell. Once inside the cell, cystine is eventually reduced to cysteine, which is critical for constructing the tripeptide GSH [13,14]. GPX4 then uses GSH as a reducing cofactor to reduce lipid hydroperoxides to their corresponding non-toxic lipid alcohols, interrupting the peroxidation chain reaction [15]. Maintaining this antioxidant system requires NADPH, which provides the reducing power to regenerate GSH and sustain GPX4 activity [16]. Thus, ferroptosis arises from excess iron availability paired with the disruption of the cellular processes that maintain lipid peroxide detoxification, including cystine uptake, GSH synthesis, and GPX4 activity.

## 3. GBM-Specific Ferroptosis Biology

GBM exhibits several intrinsic characteristics that increase its susceptibility to fer-roptosis. GBM cells exhibit “iron addiction” to support rapid proliferation, as reflected by increased expression of the iron-importing protein transferrin receptor 1 (TFR1) [17]. Additionally, the brain is naturally enriched in long-chain PUFAs, particularly docosahexaenoic acid (DHA) and arachidonic acid (AA), providing an abundance of lipids that are susceptible to oxidation [9]. GBM cells have also been demonstrated to employ several compensatory mechanisms to counter the risks incurred by their high iron demand. Overactivation of the GSH-GPX4 axis is one key example, with GPX4 providing the principal enzymatic defense against accumulating lipid hydroperoxides [18]. Alterations in lipid metabolism represent a second mechanism, as the enzymatic handling of PUFAs determines how much peroxidizable substrate is incorporated into membrane phospholipids [19]. Notably, the mesenchymal GBM subtype exhibits higher GSH levels and a broadly reinforced antioxidant program, corresponding with reduced susceptibility to ferroptotic induction [20]. GBM can also utilize the transsulfuration pathway, converting methionine-derived intermediates into cysteine to sustain GSH synthesis and GPX4 activity when extracellular cystine is limited [21]. Additionally, GPX4 expression is elevated in glioma tissues compared with normal brain and significantly increases with tumor grade, allowing high-grade tumors to suppress significant levels of ferroptotic damage [18].

Several other GBM-specific pathways that modulate sensitivity to ferroptosis have also been well-described. Acyl-coenzyme A synthetase long-chain family member 4 (ACSL4), for instance, is an enzyme that incorporates PUFAs into membrane lipids, thus generating substrates susceptible to peroxidation; relatively low ACSL4 expression in GBMs can therefore limit lipid peroxide formation and confer resistance to ferroptosis [19]. The tumor suppressor p53 offers another connection between GBM genetics and ferroptosis. Wild-type p53 can suppress SLC7A11, reducing cystine uptake and GSH pro-duction and thereby promoting ferroptosis [13]. But as p53 mutations are common in GBM, loss of this regulatory function can contribute to ferroptosis resistance. NRF2 is a transcription factor that activates genes involved in antioxidant defense and normally protects cells from oxidative stress. In TMZ-resistant GBM, increased NRF2 activity promotes expression of ABCC1, a transporter that exports GSH from cells, increasing their sensitivity to ferroptosis [22]. Non-coding RNAs provide an additional layer of regulation, with the best characterized being miR-147a, which promotes ferroptosis in GBM by sup-pressing SLC40A1, an iron-exporting protein [23].

Ferroptosis may be particularly relevant to GSCs, a malignant subpopulation capable of sustaining tumor growth and contributing to therapeutic resistance and recurrence. GSCs demonstrated higher levels of autophagy relative to differentiated glioma cells, which can be cytoprotective of the GSC population during periods of oxidative stress [24]. Furthermore, inhibition of autophagy has been shown to increase ferroptotic death in GSCs and sensitize them to TMZ [25]. More recently, CENPA, a protein involved in maintaining the stem-like state of GSCs, was shown to promote GSC survival by suppressing ferroptosis, further demonstrating that ferroptosis susceptibility varies with glioma cell differentiation [26].

## 4. Mechanisms of Radiation-Induced Ferroptosis in GBM

RT can substantially increase the oxidative burden within tumor cells. In particular, RT can increase ACSL4 expression and deplete intracellular GSH, favoring an accumulation of lipid peroxides and ferroptotic cell death [27]. Likewise, inhibition of ferroptosis has been shown to reduce radiation-induced cytotoxicity [28].

RT can also, however, trigger adaptive responses that can limit ferroptotic vulnerability. Radiation-induced oxidative stress can increase SLC7A11 and GPX4 expression, allowing surviving cells to restore their antioxidant defenses [27]. This may contribute to the emergence of radioresistant tumor populations. Interestingly, Kram et al. found that recurrent tumors exhibited increased ACSL4 and decreased GPX4 compared with matched primary tumors, suggesting that radiation and chemoradiation may select for tumors with potentially exploitable ferroptotic vulnerabilities [29].

Several mechanisms may contribute to GBM survival after RT. Zhou et al. identified gamma-interferon-inducible protein 16, IFI16, a protein associated with cellular senescence, as a mediator of radioresistance through heme oxygenase 1 (HMOX-1), a stress-response enzyme involved in heme degradation [30]. In radio-resistant GBM cells, IFI16 increased HMOX1 expression, which reduced ROS, lipid peroxidation, and available iron, thereby limiting ferroptosis. On the other hand, disrupting IFI16 restored radiosensitivity. Additionally, the long non-coding RNA POT1-AS1 promotes resistance to both RT and TMZ by stabilizing GPX4 mRNA through IGF2BP2, an RNA-binding protein that regulates mRNA stability [31].

Hypoxia, a hallmark of GBM, can limit ferroptosis as low oxygen availability limits the necessary ROS production for ferroptotic cell death. Huang et al. found that this barrier could be overcome by depleting carbonic anhydrase 9 (CA9), a hypoxia-associated enzyme that helps tumor cells adapt to the low-oxygen conditions [28]. In the study, CA9 knockdown enhanced radiation-induced ferroptosis by altering iron regulation. Using transcriptomic approaches, Hacioglu et al. recently identified a transcriptomic association between hypoxia signaling and ferroptosis-related gene signatures in GBM [32]. This association was shown to be mediated by HILPDA, a hypoxia-inducible protein involved in lipid metabolism. Further exploration of HILPDA is warranted. These papers suggest that the adaptations that allow GBM cells to survive in their microenvironment can simultaneously determine their response to radiation-induced ferroptosis.

**Figure 1:**
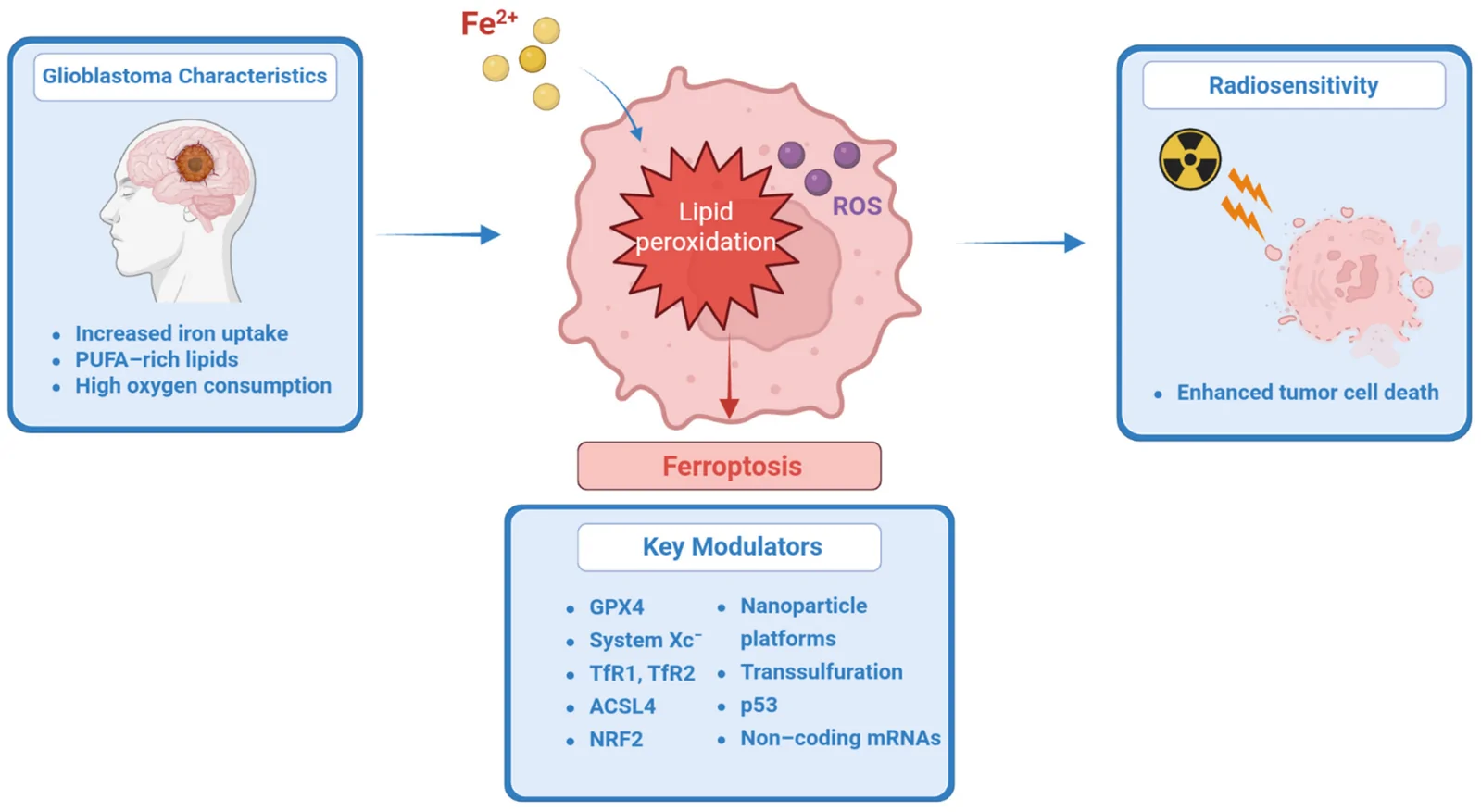
Determinants of ferroptotic radiosensitivity in glioblastoma. Left: tumor-intrinsic features that create a permissive substrate for ferroptosis, namely increased iron uptake, PUFA-rich membrane lipids, and high oxygen consumption. Center: ferrous iron (Fe^2+^) drives Fenton chemistry and ROS generation, which propagate peroxidation of membrane phospholipids; once peroxide generation exceeds the detoxifying capacity of the cell, ferroptotic death follows. Right: during radiotherapy the net consequence of this process is enhanced tumor cell killing, that is, radiosensitization. Bottom: the principal modulators that set the ferroptotic threshold in GBM, comprising antioxidant and transport machinery (GPX4, system Xc^−^, transsulfuration pathway), iron-handling proteins (TFR1, TFR2), the substrate-generating enzyme ACSL4, transcriptional and post-transcriptional regulators (NRF2, p53, non-coding RNAs), and engineered nanoparticle platforms used to deliver ferroptosis inducers across the BBB.

Collectively, these studies highlight the potential of leveraging RT-induced ferroptosis to improve GBM treatment (Figure 1). Rather than simply increasing radiation-induced oxidative stress, combining RT with ferroptosis inducers may help target both primary tumor cells and resistant populations that survive conventional radiation.

## 5. Strategies to Enhance Ferroptosis for GBM Radiosensitization

Pharmacologic approaches to enhancing ferroptosis in GBM include classical ferroptosis inducers (FINs), repurposing of FDA-approved drugs, and NRF2 inhibitors [11]. These strategies are summarized and compared in Table 1. It should be stated at the outset that most approaches discussed in this section are supported by in vitro and in vivo data only, so translational language used below should be read accordingly. There are four classes of FINs. Class I FINs include erastin and imidazole ketone erastin (IKE). These FINs inhibit SLC7A11, lowering cystine uptake and reducing intracellular cystine and GSH levels. Sulfasalazine, an FDA-approved anti-inflammatory agent, has demonstrated class I FIN activity in glioblastoma [14]. Class II FINs include RSL3, ML162, and ML210, which are small-molecule covalent GPX4 inhibitors that block ROS detoxification. Class III FINs include FIN56, among others. These FINs function by lowering GPX4 and ubiquinone stores and have shown efficacy in vivo [33]. Class IV FINs like FINO2 oxidize iron and indirectly inactivate GPX4 but have not been assessed in vivo [34]. Of these, RSL3 and sulfasalazine have demonstrated preclinical synergy with RT in gliomas [34,35].

Sulfasalazine is also the only agent in this class to have been tested clinically alongside RT in GBM. Building on the radiosurgery work above, Skeie et al. conducted a phase 1 dose-escalation trial in which 12 patients with recurrent IDH-wildtype GBM received sulfasalazine at 1.5–6.0 g/day for three days before single-fraction stereotactic radiosurgery [36]. The combination produced no dose-limiting toxicity, and MR spectroscopy showed intratumoral glutathione falling while glutathione in normal brain rose, with local control exceeding that of contemporaneous controls; a randomized phase 2 trial is planned. Sulfasalazine is separately being evaluated alongside standard chemoradiotherapy in newly diagnosed GBM [37]. Of note, these investigators framed their findings in terms of glutathione depletion and radiation-induced oxidative stress rather than ferroptosis, and neither trial measured ferroptotic cell death directly.

When repurposed, select FDA-approved drugs have shown promise as enhancers of ferroptosis or TMZ efficacy in glioma treatment. Haloperidol, a dopamine receptor 2 antagonist (DRD2) antipsychotic, was assessed in conjunction with TMZ for in vitro and in vivo GBM treatment. Through DRD2 suppression, haloperidol increased endoplasmic reticulum stress in GBM cells, leading to increased autophagy and ferroptosis. Haloperidol exhibited significant synergistic effects with TMZ, enhancing TMZ efficacy and providing a new avenue to combat TMZ resistance [38]. Glyburide, a sulfonylurea originally developed for diabetes treatment, enhanced lipid peroxidation, ferroptosis, and radiosensitivity in GBM cells in vivo and in vitro through antagonism of IFI16. In radioresistant GBM cells, IFI16 drives expression of HMOX-1, which lowers ROS, lipid peroxidation, and the labile iron pool; the resulting evasion of ferroptosis sustains radioresistance, and pharmacological inhibition of IFI16 by glyburide reverses it [30]. FTY720, also known as fingolimod, is a fungal derived sphingosine 1-phosphate receptor modulator recently shown to induce ferroptosis in GBM cells in vitro and in vivo through the AMPK-mTOR-GPX4 pathway. Delivery of FTY720 across the blood–brain barrier (BBB) was enhanced by lipid nanoparticle (LP) encapsulation. The addition of ginsenoside F2 (GF2) to these LPs reduced immunogenicity while improving GBM and BBB targeting via GLUT1 receptor binding specificity in vitro and in vivo. GF2 also demonstrated a synergistic effect with FTY720 by lowering GSH levels, providing a candidate preclinical approach for amplifying the effects of poorly bioavailable ferroptosis-inducing drugs in GBM [39]. Brusatol, a plant-derived quassinoid, is not currently FDA approved but has shown promise as a potential ferroptosis inducer through its actions as an NRF2 inhibitor. Recently, a brusatol-loaded nanoparticle (NP) system was shown to induce ferroptosis and increase the radiosensitivity of GBM cells in vitro and in vivo through the NRF2-GSH-GPX4 axis [40]. The NP system was based around a metal–organic framework (MOF) that coordinated hafnium (Hf^4+^) and iron (Fe^3+^) with a porphyrin ligand. Brusatol was loaded onto the nanoplatform and the surface was modified with transferrin-tannic acid to enhance penetration of the BBB and active GBM targeting. Upon irradiation, Hf enhanced the radiation deposition in GBM cells, resulting in upregulation of ACSL4, which upregulates ferroptosis by generating more PUFA. Simultaneously, the MOF nanoplatform increased the labile iron pool, driving an increase in Fenton reactions, and the brusatol-mediated NRF2-GSH-GPX4 axis inhibition decreased antioxidant defense. All these mechanisms synergized to induce ferroptosis in GBM cells in vitro and in vivo.

Other NP-based platforms which have demonstrated successful in vitro and in vivo BBB penetration and ferroptosis induction in GBM include a small activating RNA (saRNA) macrophage membrane (MM)-based NP. ALOX15, a lipoxygenase in the ferroptosis pathway, was successfully upregulated in the saRNA-ALOX15-MM NP system. MM coating allowed saRNA-ALOX15 NPs to avoid phagocytosis while protein modifications to the coating promoted GBM uptake. ALOX15 upregulation increased lipid peroxidation, resulting in increased ferroptosis and RT synergy in vitro and in vivo [41]. Liang et al. engineered Fe^3+^ onto red blood cell nanovesicles (RNVs) and loaded TMZ into their phospholipid bilayers [42]. The incorporated Fe^3+^ hijacks transferrin (Tf), which then leverages highly upregulated Tf receptor expression in brain microvascular endothelial cells and GSCs to simultaneously cross the BBB and target GSCs. Once taken up, the RNVs suppressed GPX4 expression and increased ferroptosis through interconversion of hemoglobin Fe^2+^ and Fe^3+^ on the RNV surface, which consumed GSH and amplified oxidative stress in the GSCs [42]. Also leveraging Fe^3+^, Liu S. et al. used Fe^3+^ NPs coordinated with luteolin and loaded with dihydroartemisinin to cross the BBB and amplify ferroptosis in GBM [43]. Using ApoE to facilitate BBB entry and GBM targeting, Li B. et al. engineered an upconversion nanoparticle (UCNP) loaded with glucose oxidase (GOx) and sorafenib (SRF) [44]. SRF, originally developed and FDA-approved for hepatocellular carcinoma, suppresses system Xc^−^ and inhibits GSH synthesis, downregulating GPX4 and inducing ferroptosis. Upon exposure to near-infrared radiation (NIR), the ApoE-UMSNs-GOx/SRF NPs exhibited a three-pronged, synergistic mechanism of ferroptosis induction: SRF initiated ferroptosis and promoted release of free Fe^2+^ from ferritin to fuel the Fenton reaction, UCNP NIR exposure regenerated Fe^2+^ from Fe^3+^ leading to more ROS formation, and GOx increased H_2_O_2_, further fueling the Fenton reaction and improving the efficiency of ferroptosis in GBM [45]. Magnetic nanoparticles (MNPs) have shown promise in drug delivery when combined with magnetic helmets to guide MNPs to the correct location. Li B et al. developed a MNP system which combined extracranial magnetic field generation with an angiopep-2 (ANG) peptide-decorated exosome (EXO) targeting system that specifically binds lipoprotein-1 receptors highly expressed on brain capillary and glioma cells [44]. This targeting MNP platform was created around a Fe_3_O_4_ core and was loaded with small interfering RNA of GPX4 and brequinar (BQR). Mechanistically, BQR sensitizes cells to ferroptosis via inhibition of dihydroorotate dehydrogenase (DHODH) which works in parallel to GPX4 in mitochondria to protect against ferroptosis. The MNP-BQR-ANG-EXO-siGPX4 system sensitized GBM cells via GPX4 and DHODH inhibition, while the Fe_3_O_4_ core released Fe^2+^, increasing the Fenton reaction and inducing ferroptosis.

Targeting genes which encode ferroptosis mediators has shown promise for GBM treatment as well. Overexpression of SLC7A11 or GPX4 mitigates ionizing radiation-induced ferroptosis, promoting radioresistance. Inhibition of SLC7A11 or GPX4 can be achieved by class I FINs or via genetic inhibition [27]. MicroRNAs (miRNAs) offer the ability to attenuate gene expression, and were recently utilized to target PPP2R5E, a critical regulatory subunit of serine/threonine protein phosphatase PP2A. MiR-19b upregulation suppresses PPP2R5E, leading to poor TMZ response. Suppression of miR-19b and subsequent PPP2R5E upregulation increased ferroptosis in GBM, contributing to TMZ-induced cytotoxicity [46]. Non-coding RNA (ncRNA) POT1-AS1 can modulate TMZ resistance through ferroptosis resistance. TMZ-resistant GBM cells overexpressed POT-AS1 and GPX4, with POT-AS1 knockdown resulting in GPX4 downregulation and increased ferroptosis. POT-AS1 knockdown sensitized GBM cells to TMZ and radiation, highlighting the role of ferroptosis in treatment resistance [31]. Exostosin glycosyltransferase (EXT2), involved in heparin sulfate synthesis, knockout resulted in radiosensitization and increased ferroptosis in an erastin-independent mechanism. EXT2 knockout increased S-adenosylmethionine (SAM) which promoted lipid oxidation and downregulated transsulfuration reactions, resulting in increased ROS and ultimately increased ferroptosis. These results highlight EXT2 as a novel target for future ferroptosis-focused therapies [47].

TMZ, the current chemotherapeutic standard of care for GBM, has also been recognized to induce ferroptotic responses. TMZ results in upregulation of divalent metal iron transporter (DMT1), resulting in increased intracellular iron levels. Further, TMZ downregulated the NRF2-HMOX-1 pathway, which is critical to antioxidant stress mitigation and autophagy. DMT1 downregulation increased GPX4, NRF2, and HMOX-1, suggesting TMZ achieves cytotoxicity through DMT1 upregulation [48]. Sehm et al. showed in rodent and human glioma cells that TMZ cytotoxicity is modulated by xCT/SLC7A11 expression and is potentiated by erastin and sorafenib, and TMZ-resistant GBM cells with elevated NRF2 and ABCC1/MRP1 become collaterally sensitive to erastin-induced ferroptosis through GSH depletion [22,49]. Targeting ferroptosis provides a therapeutic avenue for some of the most difficult GBM cell types to eliminate, including IDH-wt cells and GSCs. When combined with RSL3, a class II FIN, TMZ resulted in GBM growth suppression and ferroptosis induction regardless of IDH status [50]. After autophagy inhibition by quinacrine, a derivative of hydroxychloroquine, TMZ treatment induced ferroptosis of GSCs [25]. TMZ cytotoxicity was also significantly increased upon overexpression of transferrin receptor 2 (TFR2) in glioma cells. TFR2 promotes iron entry to cells, driving the Fenton reaction and synergizing with TMZ-promoted ferroptosis. As such, TRF2 expression levels could serve as a prognosticator for future ferroptosis-based treatments in gliomas [51]. TMZ-resistant GBM cells exhibited high NRF2 levels, which upregulated ATP binding cassette subfamily C member 1/multidrug resistance-associated protein 1 (ABCC1/MRP1). Increased NRF2 and high ABCC1/MRP1 levels contributed to GSH depletion after erastin-mediated system Xc^−^ inhibition, promoting ferroptosis. Collateral ferroptosis sensitivity may be achieved as GBM cells upregulate NRF2 to efflux TMZ via increased MRP1, while high MRP1 depletes GSH and sensitizes TMZ-resistant cells to ferroptosis induction [13].

**Table 1:** Strategies for ferroptosis-mediated radiosensitization in GBM. Entries are grouped as pharmacologic ferroptosis inducers, repurposed agents, NPs, and genetic approaches, in the order discussed in the text.

Strategy/Agent	Molecular Target and Mechanism	Highest Level of Evidence	Evidence in Combination with RT (or TMZ)	Ref.
Erastin, imidazole ketone erastin (Class I FIN)	SLC7A11 (system Xc^−^) inhibition; reduced cystine uptake and GSH synthesis	Glioma cell lines, including TMZ-resistant lines	Ferroptosis induction and collateral sensitivity in TMZ-resistant cells; not tested with RT in GBM	[11,22]
Sulfasalazine (Class I FIN; FDA-approved)	System Xc- blockade with GSH depletion	Rodent and human glioma lines; orthotopic xenograft; two completed clinical trials	Radiosensitization with gamma knife radiosurgery in vivo. Phase 1 trial with single-fraction radiosurgery in recurrent GBM: no dose-limiting toxicity, intratumoral glutathione fell on MR spectroscopy while normal brain rose, local control better than contemporaneous controls; randomized phase 2 planned. An earlier monotherapy trial without RT was terminated at interim analysis for toxicity	[14,36,52,53]
RSL3, ML162, ML210 (Class II FINs)	Covalent GPX4 inhibition blocking lipid hydroperoxide detoxification	Glioma lines; patient-derived early-passage cultures and surgical slice cultures	RSL3 sensitizes glioma cells to ionizing radiation by suppressing TGM2-dependent DNA repair and EMT; selective depletion of quiescent astrocyte-like cells in patient-derived material	[35,50,54]
FIN56 (Class III FIN)	Depletion of GPX4 and ubiquinone; TFEB-dependent lysosomal membrane permeabilization	GBM lines with in vivo confirmation	Ferroptosis induction; not tested with RT	[33]
FINO2 (Class IV FIN)	Iron oxidation with indirect GPX4 inactivation	In vitro only	No in vivo or RT data	[34]
Haloperidol (repurposed DRD2 antagonist)	DRD2 suppression, ER stress, autophagy-dependent ferroptosis	GBM lines; in vivo	Synergy with TMZ; not tested with RT	[38]
Glyburide (repurposed sulfonylurea)	IFI16 antagonism reversing HMOX1-dependent ferroptosis evasion	Radioresistant GBM cells; in vivo	Radiosensitization in vitro and in vivo	[30]
FTY720 (fingolimod) in ginsenoside F2-modified liposomes	AMPK-mTOR-GPX4 axis; GLUT1-mediated BBB transit; GSH lowering by GF2	GBM lines; orthotopic in vivo	Ferroptosis induction and improved targeting; RT not tested	[39]
Brusatol-loaded Hf/Fe metal–organic framework	NRF2-GSH-GPX4 inhibition plus high-Z radiation dose deposition and labile iron expansion	GBM lines; orthotopic in vivo	Radiosensitization in vitro and in vivo	[40]
saRNA-ALOX15 macrophage-membrane nanoparticle	ALOX15 upregulation increasing lipid peroxidation; phagocytosis avoidance	GBM lines; in vivo	Ferroptosis and RT synergy in vitro and in vivo	[41]
Fe^3+^ red blood cell nanovesicles loaded with TMZ	Transferrin hijacking for BBB transit and GSC targeting; GPX4 suppression and GSH consumption	Glioma stem cells; orthotopic in vivo	Tumoricidal activity and recurrence inhibition with TMZ; RT not tested	[42]
Luteolin-coordinated Fe^3+^ nanoplatform with dihydroartemisinin	Fenton amplification after BBB penetration	GBM lines; in vivo	Ferroptosis amplification; RT not tested	[43]
ApoE-UCNP-GOx/sorafenib nanoparticle	System Xc- inhibition, ferritin iron release, NIR-driven Fe^2+^ regeneration, GOx-generated H_2_O_2_	GBM lines; in vivo	Three-pronged ferroptosis induction under near-infrared, not ionizing, radiation	[45]
MNP-BQR-ANG-EXO-siGPX4 magnetic exosome platform	Parallel GPX4 and DHODH inhibition with Fe_3_O_4_-derived Fe^2+^ release; magnetically guided delivery	GBM lines; in vivo	Ferroptosis induction; RT not tested	[44]
Genetic inhibition of SLC7A11 or GPX4	Loss of cystine import or of lipid hydroperoxide detoxification	Tumor lines including glioma; in vivo	Overexpression confers radioresistance; inhibition restores radiation-induced ferroptosis	[27]
miR-19b suppression (PPP2R5E restoration)	Restoration of the PP2A regulatory subunit PPP2R5E	GBM cells	Increased ferroptosis and TMZ-induced cytotoxicity	[46]
POT1-AS1 knockdown	Destabilization of GPX4 mRNA through IGF2BP2	TMZ- and RT-resistant GBM cells	Resensitization to both TMZ and radiation	[31]
EXT2 knockout	SAM accumulation with downregulated transsulfuration, raising ROS	GBM cells	Radiosensitization through an erastin-independent mechanism	[47]
CA9 knockdown	Relief of hypoxic restraint on ferroptosis with altered iron regulation	Hypoxic glioma cells	Enhanced radiation-induced ferroptosis and radiosensitivity	[28]
Ferroptosis inducers combined with immunotherapy (ICB, NK-exosome/RSL3 hybrids, Cu3PdN nanozymes, hemoglobin nanoplatforms, CuS liposomes)	Coupling tumor ferroptosis to DC maturation, CD8^+^ T cell priming, M2-to-M1 repolarization and PD-L1 modulation	Orthotopic GBM models	Antitumor synergy with checkpoint blockade; combination with RT largely untested	[55,56,57,58,59,60]

## 6. Ferroptosis, Radiotherapy, and the GBM Immune Microenvironment

GBMs exhibit one of the most immunosuppressive tumor microenvironments (TMEs) in oncology, and ferroptosis has been shown to be a key modulator of immune cell composition and function in the glioma milieu [11,55,61]. Because radiotherapy itself remodels this compartment, the immunologic consequences of ferroptosis are considered here specifically as they bear on the radiation response, rather than as a general account of ferroptosis in tumor immunity.

Activated clusters of CD8^+^ T cells can directly trigger ferroptosis in tumor cells by secreting IFNγ, which suppresses SLC7A11 and depletes intracellular GSH, leaving cancer cells vulnerable to lipid peroxidation [62]. RT and immunotherapy achieve this suppression through independent but synergistic pathways, meaning even limited CD8^+^ T cell activity in GBM can amplify radiation-induced ferroptosis [63]. IFNγ released by CD8^+^ T cells also increases the incorporation of PUFAs into tumor cell membranes via ACSL4 upregulation, further sensitizing GBM cells to ferroptotic death [64,65,66]. This axis has been validated in orthotopic brain tumor models that use engineered bacterial outer membrane vesicles (OMVs) to enhance CD8^+^ T cell infiltration. Chen et al. developed these OMVs through a genetic modification of *Escherichia coli* to efficiently cross the BBB and specifically target tumor cells in vitro and in vivo. Increased CD8^+^ T cell infiltration and subsequent IFNγ-mediated ferroptosis were observed both in vitro and in vivo, including in TMZ-resistant cells [67].

Ferroptotic tumor cells release damage-associated molecular patterns (DAMPs), in-cluding high-mobility group box 1 (HMGB1), adenosine triphosphate (ATP), and calre-ticulin (CRT), that activate innate immunity by promoting dendritic cell (DC) maturation, macrophage repolarization, and enhanced CD8^+^ T cell priming [66,68,69,70,71]. However, the immunogenicity of ferroptosis appears to be time-dependent. Efimova et al. demonstrated that early ferroptotic GL261 glioma cells induced bone marrow-derived dendritic cell (BMDC) maturation and conferred vaccination-like protection against the GL261 cells in immunocompetent mice, while late-stage ferroptotic cells lost this capacity [72]. Janssens et al. further showed the important caveat that late ferroptotic cells resist DC engulfment, and that prostaglandin E2 (PGE2) and oxidized lipids released during prolonged ferroptosis can impair antigen-presenting cell (APC) function [73].

In a glioma-specific study, DCs loaded with ferroptotic tumor cell lysates conferred protection against intracranial glioma in mice, generating robust effector memory T cell responses dependent on CRT and ATP signaling rather than HMGB1-Toll-like receptor 4 (TLR4) pathways [74]. These findings point to a positive feedback loop in which radiation-induced ferroptosis releases DAMPs, activates DCs, primes CD8^+^ T cells to secrete IFNγ, and IFNγ in turn drives further tumor ferroptosis [75,76].

Tumor-associated macrophages (TAMs) also play a central role in modulating ferroptosis susceptibility within the GBM microenvironment. Sahu et al. demonstrated that TAMs promote ferroptosis resistance in GBM cells by stimulating the release of iron-loaded extracellular vesicles from GL261 cells via tumor necrosis factor alpha (TNFα) signaling; inhibiting this vesicle pathway resensitized GBM to RSL3-induced ferroptosis [77]. Complementing this, Guo et al. showed that M2-polarized macrophages confer ferroptosis resistance to GBM cells by suppressing nuclear receptor coactivator 4 (NCOA4) and enhancing ferritin heavy chain 1 (FTH1) expression, reducing the labile iron pool. Furthermore, co-implantation of M2 macrophages abrogated erastin’s antitumor effect in vivo [78]. Conversely, inducing ferroptosis in GBM cells can reshape TAM polarization. Zhao et al. showed that nicotinamide phosphoribosyltransferase (NAMPT) inhibition induced ferroptosis in GL261 cells and promoted M2-to-M1 macrophage repolarization, enhancing prophylactic vaccination efficacy in orthotopic GBM models [79]. Xu et al. identified ferroptosis-related risk gene signatures in GBM that correlated with skewed M1-to-M2 ratios and worse prognosis, further emphasizing the bidirectional nature of the TAM-ferroptosis axis [80]. Qian et al. provided a broader framework for understanding this immunometabolic crosstalk, highlighting how iron flux, PUFA availability, and redox balance between TAMs and ferroptotic cancer cells jointly determine the net immunological outcome [81].

A major challenge is that ferroptosis is not tumor-selective. Intra-tumoral CD8^+^ T cells are more susceptible to ferroptosis than cancer cells, and non-selective inhibition of GPX4 or SLC7A11 can deplete natural killer (NK) cells, macrophages, and effector T cells alongside tumor cells [82,83]. Additionally, ferroptosis of tumor-associated neutrophils re-leases oxygenated lipids that paradoxically suppress T cell function [84]. In GBM, ferroptosis is the most enriched form of programmed cell death, and high ferroptosis gene signatures correlate with immunosuppression and poor outcomes through TAM-mediated mechanisms [56]. A ferroptosis-resistant myeloid-derived suppressor cell (MDSC) subcluster has been identified in peri-necrotic GBM niches, driving mesenchymal progression and resistance to anti-programmed cell death protein 1 (PD-1) therapy [85]. Ferroptotic cells additionally release immunosuppressive mediators such as PGE2, and HMGB1-driven signaling can upregulate programmed death-ligand 1 (PD-L1) on tumor cells [83,86]. Blanket ferroptosis induction in GBM therefore risks killing tumor cells while simultaneously crippling the immune effectors needed for durable control [87].

Several emerging approaches aim to selectively induce tumor ferroptosis while preserving or enhancing immune function. Combining ferroptosis inducers with immune checkpoint blockade (ICB) leverages synergistic suppression of SLC7A11 while restoring CD8^+^ T cell function; preclinical GBM models have shown synergistic antitumor effects with this approach [55,56,61]. Nanoplatform-based strategies are particularly promising for GBM given the BBB. Hao et al. developed NK cell exosome-RSL3 hybrid nanovesicles that established a positive ferroptosis-immunotherapy feedback cycle in glioma, with IFNγ and TNFα released from activated immune cells stimulating DC maturation and further GPX4 inactivation in tumor cells [57]. Chen et al. engineered Cu_3_PdN nanozymes that depleted cholesterol and downregulated PD-L1 while triggering ferroptosis and immunogenic cell death (ICD) with CRT exposure, DC maturation, and CD8^+^ T cell infiltration in orthotopic GBM [58]. Tao et al. designed a hemoglobin-based nanoplatform that induced ferroptosis while reprogramming M2 macrophages to M1 via the haptoglobin pathway to create an immunostimulatory TME. The effects of this nano-platform were shown to synergize with anti-PD-L1 therapy in vivo [59]. Zhou et al. demonstrated that chitosan-coated copper sulfide liposomes induced ferroptosis-mediated ICD in glioma, achieving notable cytotoxic T cell infiltration and DC maturation [60]. Finally, ferroptosis-armed DC vaccines bypass systemic ferroptosis induction entirely, using ferroptotic tumor lysates to generate robust antitumor immunity without collateral immune cell damage [74]. Taken together, these data indicate that the lack of tumor selectivity of ferroptosis is unlikely to be solved by a systemically delivered inducer. The more tractable strategies couple ferroptosis induction to a tumor-restricted trigger: receptor-targeted or membrane-camouflaged nanocarriers that accumulate preferentially in GBM, platforms that confine lipid peroxidation to the irradiated volume, exploitation of the higher labile iron pool and ACSL4 expression of tumor cells relative to neurons, and ex vivo approaches such as ferroptosis-armed DC vaccines that avoid systemic induction altogether.

## 7. Radiation-Induced Brain Injury and Ferroptosis

The same ferroptotic processes that make radiation effective against GBM may also cause collateral damage to the surrounding normal brain. Radiation-induced brain injury remains a major complication of cranial RT, manifesting as cognitive decline, cerebrovascular dysfunction, and diminished quality of life [88]. As ferroptosis-based radiosensitization strategies advance, understanding and mitigating ferroptosis in healthy brain tissue has become an urgent priority.

The brain is intrinsically vulnerable to ferroptosis. Its high PUFA content, elevated baseline iron levels, and substantial oxygen consumption create a permissive environment for the lipid peroxidation that drives ferroptotic cell death [89,90,91]. Neurons are particularly susceptible because they rely on neighboring astrocytes for antioxidant support, including supply of cysteine for GSH synthesis via system Xc^−^, meaning that any disruption of this metabolic partnership leaves neurons defenseless [89,91]. Once initiated, ferroptotic injury can propagate across cell types, from microglia to astrocytes to neurons, amplifying damage beyond the initially affected population [89]. Huang et al. reviewed ferroptosis across central nervous system injuries broadly, noting that emerging diagnostic tools such as iron-sensitive MRI may eventually allow clinicians to detect ferroptotic damage in irradiated brain tissue [92].

Preclinical studies have confirmed that whole brain radiation triggers ferroptosis in hippocampal neurons, characterized by iron accumulation, depletion of GPX4, and downregulation of SLC7A11, with concurrent upregulation of the pro-ferroptotic enzyme ACSL4 [93]. Su et al. emphasized that the cooperation between radiation and ferroptosis enhances tumor killing but simultaneously increases risk to normal tissues [94]. Autophagy further muddles the picture: while it can be protective by clearing damaged cellular components, it can also worsen ferroptosis by releasing stored iron through ferritinophagy [88,93,95,96]. Chen et al. showed that mixed carbon ion–-photon radiotherapy (CIRT), which utilizes heavy ions to deliver precisely localized charged particle therapy, pro-motes ferroptosis in GBM by enhancing this mechanism of ferritinophagy [97]. The hope of CIRT is that its capacity for precision delivery of therapy ultimately minimizes collateral damage to healthy surrounding tissue. Further studies are thus warranted to investigate the potential of this strategy.

Several neuroprotective strategies have shown preclinical promise. Ferrostatin-1 and liproxstatin-1, established ferroptosis inhibitors, have been effective in reducing radiation-induced brain injury in animal models [98,99]. Another major approach to this problem among investigators has been the selective activation of antioxidant defense pathways. Shi et al. showed that genetic modulation of NRF2 signaling protected neurons from radiation-induced ferroptosis, while Jiajia et al. demonstrated that Maresin-1 alleviated brain injury after irradiation through a process mediated by NRF2 and retinoic acid receptor-related orphan receptor alpha (RORα) [100,101]. Repetitive transcranial magnetic stimulation has also emerged as a non-pharmacological intervention that restores GPX4 and SLC7A11 expression while suppressing ACSL4, ultimately leading to anti-ferroptotic effects that improve cognition in irradiated animals [102,103].

**Figure 2:**
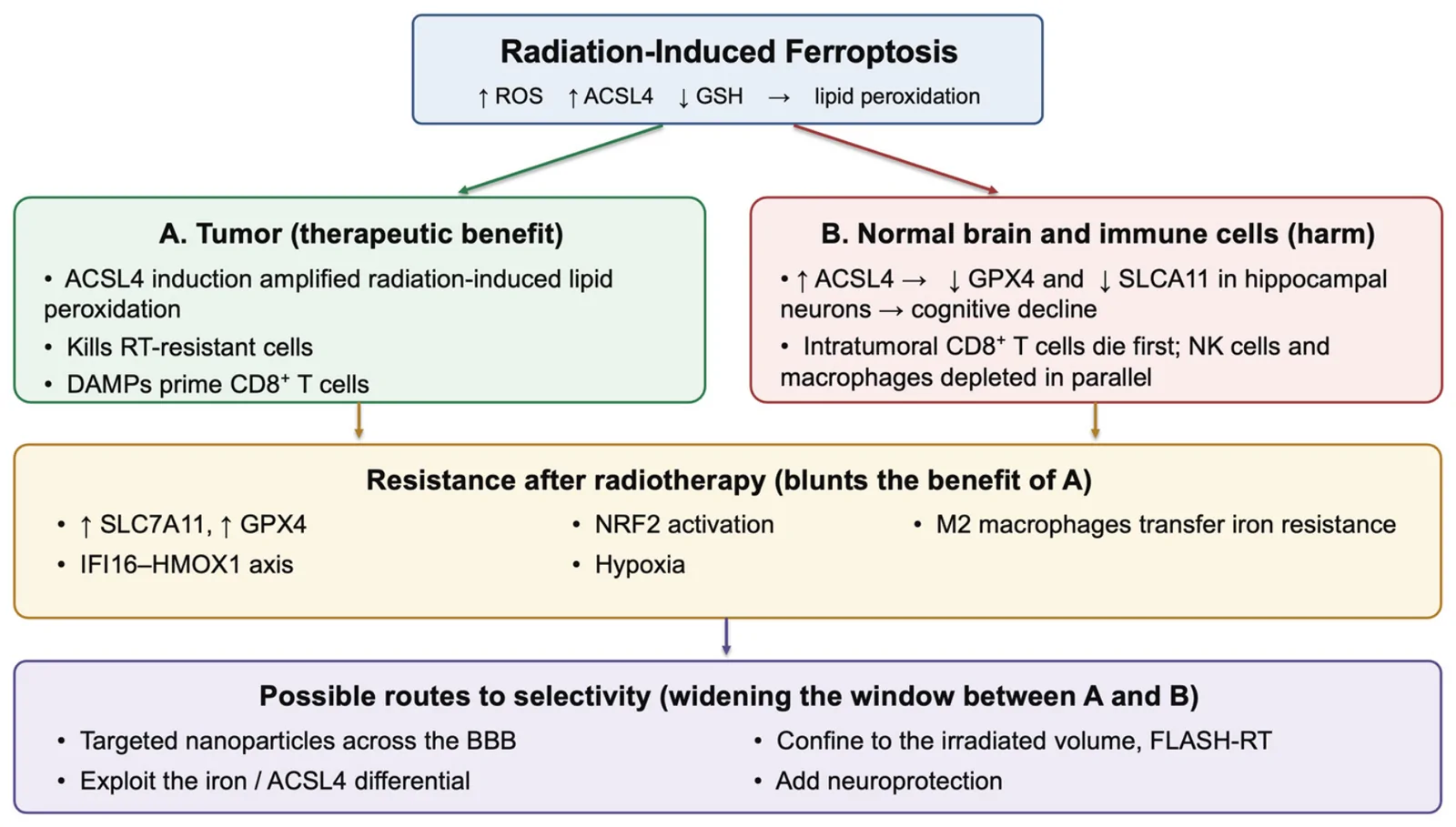
The dual role of radiation-induced ferroptosis in GBM and the routes to selectivity. RT delivered to the tumor volume raises ROS, induces ACSL4, and depletes GSH, driving phospholipid peroxidation in every cell within the irradiated field. (A) In the tumor compartment this is therapeutically favorable: an iron-avid, PUFA-rich phenotype supplies abundant peroxidation substrate, ferroptosis reaches populations that survive conventional radiotherapy, and DAMP release couples tumor death to CD8^+^ T cell priming and IFN-gamma-driven amplification. (B) In normal brain and in infiltrating immune cells the same chemistry is injurious: neurons depend on astrocytic cysteine supply through system Xc^−^ and sit in a high-iron, PUFA-rich organ, whole-brain irradiation induces ACSL4 while depleting GPX4 and SLC7A11 in hippocampal neurons, and intratumoral CD8^+^ T cells are more ferroptosis-prone than the tumor cells they are meant to kill. Center: adaptive programs engaged after irradiation that blunt the benefit in (A), including SLC7A11/GPX4 induction, NRF2 activation, hypoxia signaling, the IFI16-HMOX1 axis, and iron-handling resistance transferred by M2-polarized TAMs. Bottom: strategies proposed to widen the therapeutic window between (A) and (B).

The central therapeutic challenge is selectivity: inducing ferroptosis in tumor cells while protecting healthy brain. Nanoparticle-based delivery platforms that cross the BBB and preferentially accumulate in glioblastoma represent one of the most actively investigated strategies [41,43,104,105,106]. GBM cells differ from normal neurons in their iron metabolism, ACSL4 expression, and redox balance, providing an exploitable therapeutic window [107]. Huang et al. designed a dual-action nanoparticle system that generates a protective compound in normal cells while permitting unimpeded ferroptosis in tumor cells, leveraging inherent enzymatic differences between the two [108]. Koppula et al. and Emmanuel et al. identified antioxidant resistance pathways selectively active in certain tumors but not in normal brain, offering additional targets for achieving differential ferroptosis sensitivity [109,110]. Pairing tumor-targeted ferroptosis inducers with systemic neuroprotective agents remains a promising but largely unexplored combinatorial strategy [70,91,100]. The opposing consequences of radiation-induced ferroptosis in tumor and in normal brain and immune cells, the adaptive programs that restrain the tumor-directed effect, and the strategies proposed to separate the two are summarized in Figure 2.

## 8. FLASH Radiotherapy in GBM

FLASH radiotherapy (FLASH-RT), which delivers radiation at ultra-high dose rates exceeding 40 Gy/s, has generated considerable interest in neuro-oncology due to its potential to spare normal brain tissue while maintaining antitumor efficacy. Multiple preclinical GBM studies have demonstrated that FLASH-RT and conventional-rate radiotherapy (CONV-RT) are equally effective in controlling tumor growth. Montay-Gruel et al. showed equivalent tumor growth delay across multiple FLASH-RT regimens in an orthotopic GBM mouse model compared to CONV-RT, while Liljedahl et al. supported this equivalence in fully immunocompetent GBM-bearing rats with long-term follow-up [111,112]. A systematic analysis by Böhlen et al. across 66 preclinical datasets also broadly supported this equivalence, though the authors noted heterogeneity in growth assays and relatively short follow-up periods as important limitations [113].

The principal advantage of FLASH-RT lies in neurocognitive preservation. FLASH-RT has been shown to produce long-term cognitive benefits that may be driven by a reduction in ROS-induced hippocampal dendritic spine loss and neuroinflammation [114,115]. These findings have been upheld by ex vivo MRI studies demonstrating preserved brain microstructure after FLASH delivery [116]. Beyond photon-based approaches, proton FLASH-RT has also shown promise for GBM. Iturri et al. demonstrated that proton FLASH-RT preserved memory function in orthotopic glioma-bearing rats while simultaneously stimulating an effective lymphoid immune response, a dual benefit that is particularly relevant to the immunosuppressive GBM microenvironment [117]. He et al. further demonstrated time-dependent alterations in brain metabolites and gut microbiota following whole-brain FLASH-RT versus CONV-RT, suggesting systemic effects that extend beyond the irradiated field [118].

Despite these encouraging findings, significant limitations have emerged as well. A treatment-planning study by Hörberger et al. showed that FLASH-RT provided no net sparing benefit for GBM or head-and-neck tumors, with positive net sparing observed only in breast and prostate cancers [119]. This result might be attributable to GBM not meeting the condition where the tumor α/β ratio is lower than that of the surrounding normal tissue, leaving normal brain more vulnerable to radiation damage at doses proven to be therapeutic for GBM [119]. Additionally, Williams et al. reported that whole-brain proton FLASH-RT at 18 Gy in rats did not reduce cognitive toxicity compared to conventional dose rates, directly challenging the neurocognitive-sparing narrative [120]. Vozenin et al., in a comprehensive Nature Reviews Cancer perspective, emphasized that the mechanisms underlying the FLASH effect remain incompletely understood, citing hypotheses around long-lived protein oxidation and matrix rigidity, and that evidence of a generalized FLASH effect in humans is still lacking [121]. Early clinical trials of FLASH-RT such as FAST-01 have demonstrated safety, but are limited to superficial lesions like skin metastases, with no brain tumor-specific clinical data yet available.

Perhaps the most compelling emerging link is between FLASH-RT and ferroptosis. Vilaplana-Lopera et al. first proposed that differential ferroptosis susceptibility explains the FLASH effect, hypothesizing that FLASH-RT increases ferroptosis in iron-rich tumors while sparing iron-poor normal tissues [107]. The same group subsequently provided experimental evidence demonstrating that FLASH-RT selectively increases lipid peroxidation and ferroptosis markers in tumor tissue but not normal tissue, and that a high-iron diet abrogated this protective differential. This strongly suggests that iron content is a key determinant of the FLASH effect [122]. Zhang et al. complemented this by showing that FLASH sparing involves activation of the NRF2 antioxidant pathway with reduced lipid peroxidation, subject to a critical total-dose threshold, further tying the FLASH effect to redox and ferroptosis defense mechanisms [123]. Tang et al. have recently reviewed translational strategies combining FLASH-RT with organelle-targeted radiosensitization in GBM, though their focus on endoplasmic reticulum stress pathways is inherently distinct from the ferroptosis-centered framework presented here [124]. While more prospective studies are needed and this proposal remains hypothesis-generating, these data combined raise the intriguing possibility that combining FLASH-RT with ferroptosis-inducing agents could amplify GBM treatment outcomes while possibly sparing normal brain tissue.

## 9. Ferroptosis and Radiosensitization in Other Cancers

Evidence from other radioresistant tumors is considered here for a specific purpose: to establish which features of ferroptosis-mediated radiosensitization generalize across tumor types, and, by contrast, which obstacles are particular to GBM and must be solved before the approach can be translated to the brain. Ferroptosis-RT synergy in vivo and in vitro has been explored in other treatment-resistant cancers, such as non-small cell lung cancer (NSCLC), triple-negative breast cancer (TNBC), oral squamous cell carcinoma (OSCC), nasopharyngeal carcinoma (NPC), esophageal squamous cell cancer (ESCC), and colorectal cancer (CRC). In NSCLC, erastin-mediated GPX4 downregulation and GSH depletion led to increased RT sensitization, suggesting GSH levels may potentiate ferroptosis-mediated RT response [125]. Further implicating ferroptosis as a crucial RT response mediator, NRF2 inhibition in NSCLC reversed radioresistance, increased ferroptosis, and promoted RT sensitivity [126]. In TNBC, RSL3 loaded NPs increased RT sensitivity, downregulated GPX4 and SLC7A11, and promoted ferroptosis [127]. Other pathways which achieved ferroptosis-RT synergy in TNBC included using iron saturated lactoferrin and hesperetin mediated GPX4 downregulation [128,129]. Co-treatment with hyperbaric oxygen and RT in OSCC resulted in decreased GPX4 and an increase in ferroptosis-mediated cytotoxicity in previously RT resistant cells [130]. NPC RT-ferroptosis synergy has been demonstrated to occur through SLC7A11 knockdown and GSH S-transferase mu 3-mediated GPX4 suppression in vitro [131,132]. In ESCC, NRF2-induced SLC7A11 expression promoted RT resistance in vitro, while SLC7A11 knockdown restored RT sensitivity suggesting that the observed RT resistance may have been achieved through inhibition of ferroptosis. Patients with high NRF2 and SLC7A11 expression levels exhibited poor RT response and the shortest overall survival [133]. Sulfasalazine-induced ferroptosis synergized with RT in CRC, but only under hypoxic conditions. Fang et al. found RSL3-mediated ferroptosis induction restored RT sensitivity in RT resistant CRC lines and identified NUPR1 upregulation as an inhibitor of ferroptosis [134]. These results suggest that across multiple RT resistant cancer types, ferroptosis is a viable strategy for re-sensitization.

Compared with other tumor types, GBMs have several inherent characteristics that limit their susceptibility to ferroptosis induction, including the BBB, therapy-resistant GSCs, and a dynamic immunosuppressive TME. The heterogeneous BBB imposes limits on FIN delivery; however, as previously noted in this review, nanoplatforms are showing increasing success in precision drug delivery across the BBB and to the tumor itself. PUFAs are highly concentrated in the neocortex, providing ample substrate for peroxidation and ROS [11]. While the high iron and PUFA content of the brain increases the intrinsic vulnerability of GBM cells to lipid peroxidation, what limits therapeutic gain is that the GBM cells actively regulate how much of that substrate is exposed at the cell membrane level, and that the same brain features render adjacent normal tissue vulnerable to the identical insult. GBM cells with CDKN2A deletions exhibit reduced PUFA sequestration and increased ferroptotic susceptibility [135]. GBM cells can exhibit increased intracellular iron levels, dysregulation of iron metabolism, and impaired iron storage [55]. The heterogeneous cell environment of a GBM includes self-renewing, highly resistant, and multi-potent GSCs, which have posed significant therapeutic challenges. Among this aggressive subpopulation, ferroptosis was proven to be vital in increasing susceptibility to TMZ [25]. The immunosuppressive microenvironment of GBM comprises TAMs, CD4^+^/CD8^+^ T cells, and Treg cells which all participate in iron and metabolite exchange with glioma cells. As such, GBM susceptibility to ferroptosis is highly dynamic and dependent on crosstalk within this diverse cellular milieu [11]. The comparison therefore supports a specific conclusion rather than a general one: the ferroptosis-RT interaction itself is not GBM-specific, and the mechanistic logic established in NSCLC, TNBC, OSCC, NPC, ESCC, and CRC carries over to glioma. What is GBM-specific is the combination of a highly peroxidizable, iron-rich organ shared with the tumor and a physical delivery barrier, which is why progress in GBM depends far more on selective delivery than it does in extracranial disease.

## 10. Biomarkers and Clinical Translation

The same molecular features that make ferroptosis attractive in GBM are also a source of candidate biomarkers for predicting which tumors are most likely to respond to RT. The core sensitivity switch is the balance between pro-ferroptotic and protective machinery: ACSL4, which builds lipids that act as radiation-induced peroxidation substrate, versus the antioxidant defenses GPX4 and SLC7A11. Ionizing radiation induces ACSL4 while also upregulating SLC7A11 and GPX4 as an adaptive resistance response, and higher tumor ferroptosis correlates with better RT response and survival, making these three proteins the most supported candidate radiosensitivity markers currently available [27]. Prospective validation of these markers is needed to reliably predict RT response in patients with GBM.

GBMs that recur after chemoradiation show falling GPX4 and rising ACSL4, suggesting relapsed disease is more vulnerable to ferroptosis and that these markers shift with treatment [29]. Long non-coding RNA-driven GPX4 regulation (for example, the POT1-AS1/IGF2BP2/GPX4 axis) links ferroptosis suppression directly to combined TMZ and radiation resistance, adding candidate transcript-level markers [31]. Ferritin heavy and light chains have separately been tied to both prognosis and TMZ resistance [50].

Evidence generated in patient-derived material, rather than in long-established cell lines, is the most informative for translation though remains limited. Banu et al. showed that pharmacologic GPX4 inhibition selectively depleted quiescent, astrocyte-like glioma cells in patient-derived early-passage cultures and in slice cultures prepared directly from surgical GBM specimens, identifying a cell state rather than a whole tumor as the unit of ferroptotic vulnerability and suggesting that the relevant biomarker may be a state signature rather than a bulk expression level [54]. Clinical experience with ferroptosis-relevant but non-selective approved drugs is more cautionary. Sulfasalazine, a system Xc^−^ inhibitor with clear preclinical radiosensitizing activity in glioma [52], was evaluated in a randomized phase 1/2 study in recurrent high-grade glioma that was terminated at interim analysis because of frequent grade 1–3 and four grade 4 adverse events without radiographic response [53]. The subsequent phase 1 trial combining sulfasalazine with stereotactic radiosurgery was, by contrast, well tolerated and demonstrated target engagement in patients: intratumoral glutathione fell on MR spectroscopy while glutathione in normal brain rose, and local control exceeded that of contemporaneous controls treated with radiosurgery alone [36]. This is the first human evidence that the system Xc^−^ pathway can be modulated selectively within the tumor during radiotherapy, and it also indicates a practical route around the sampling problem described below, since the readout was obtained non-invasively by imaging rather than by biopsy. The limitations are present: the trial was small, non-randomized and not powered for efficacy, and glutathione depletion is an upstream surrogate rather than a measure of ferroptotic cell death. No prospectively validated predictive ferroptosis biomarker yet exists for clinical GBM radiotherapy, and demonstrating on-target ferroptosis in human tumor tissue remains a prerequisite for interpreting any future efficacy signal, and a more immediate priority than further computational signature development.

Beyond single proteins, numerous ferroptosis-related gene signatures stratify glioma and GBM patients into prognostic risk groups, including multi-gene messenger RNA models, hypoxia-linked ferroptosis signatures, and non-coding RNA (lncRNA/circRNA) signatures [136,137,138]. These consistently associate high-risk, ferroptosis-suppressed phenotypes with worse survival and a more immunosuppressed microenvironment, though they remain retrospectively and computationally derived.

Established clinical glioma biomarkers intersect meaningfully with ferroptosis. IDH mutation, through the oncometabolite D-2-hydroxyglutarate, lowers GPX4 and depletes GSH, sensitizing mutant cells to ferroptosis. This is consistent with the greater ra-dio/chemosensitivity long observed in IDH-mutant tumors [139]. O6-methylguanine-DNA methyltransferase (MGMT) promoter methylation, the key predictor of TMZ benefit, is relevant because TMZ itself has been reported to induce ferroptotic responses, and ferroptosis inducers can resensitize resistant cells. Ferritin chain expression also correlates with MGMT status, linking the ferroptosis axis to alkylator response [48,50].

Translation of these biomarkers faces substantial hurdles. No validated, prospec-tively tested ferroptosis biomarker yet exists for clinical GBM RT, and existing signatures are limited by retrospective design, cohort-specific overfitting, and lack of standardized assays [140,141]. Marked intratumoral and intertumoral heterogeneity complicates any single-marker strategy, and the BBB constrains biomarker sampling, driving interest in circulating markers (e.g., malondialdehyde, 4-hydroxynonenal, exosomal ferroptosis transcripts) and in NP platforms that both cross the BBB and report on ferroptosis susceptibility [61,140,141]. Prospective, assay-standardized, biomarker-guided studies remain the critical missing step.

## 11. Challenges, Open Questions, and Future Directions

The central obstacle to exploiting ferroptosis in GBM is that ferroptotic sensitivity is not a fixed tumor property but a context-dependent state that varies across and within tumors. Molecular subtypes differ markedly: mesenchymal GBM cells mount a stronger antioxidant defense and resist ferroptosis induction due to higher GSH and upregulated protective genes, whereas proneural cells are comparatively vulnerable [20]. GBMs are also spatially heterogeneous in their susceptibility to ferroptosis induction, with single-cell and spatial-transcriptomic work identifying ferroptosis-resistant myeloid niches and M2-polarized TAMs that transfer ferroptosis resistance to adjacent tumor cells via iron-handling reprogramming. This particular transferred resistance program has been shown to independently predict poor survival and reduce response to checkpoint blockade [78,85]. Any ferroptosis-based strategy must therefore contend with the fact that some compartments of a given tumor will be intrinsically refractory, arguing for combinatorial induction and biomarker-based patient stratification rather than a single-target approach [61,142]. It is worth separating the two forms of heterogeneity because they call for different solutions. The dominant one is context dependency: ferroptotic susceptibility tracks with molecular subtype, differentiation state, iron handling, local oxygen tension, and the composition of the surrounding myeloid compartment, and therefore varies between tumors and between regions of the same tumor at a single time point. Superimposed on this is a slower temporal component, in that chemoradiation itself shifts the ACSL4/GPX4 balance such that recurrent tumors are, on average, more ferroptosis-primed than their matched primaries [29]. Context dependency argues for spatially informed stratification and selective delivery; the temporal shift argues that the recurrent, post-chemoradiation setting may be the more favorable one in which to test ferroptosis-directed radiosensitization first.

Dietary modulation of ferroptosis is another unexplored opportunity to potentially enhance radiation-induced ferroptotic cell death. Dietary interventions that alter iron availability, glutathione metabolism, or lipid composition may increase tumor sensitivity to radiation. Defining how these metabolic strategies can be safely integrated with RT represents an important direction for future research [143].

Hypoxia is a related and unresolved problem that undermines RT. The hypoxic GBM core drives ferroptosis resistance largely through hypoxia-inducible factor 1-alpha-dependent metabolic adaptations that sustain antioxidant capacity [143,144,145,146]. An intriguing counterpoint is that FLASH-RT retains antitumor efficacy under acute hypoxia in GBM models, unlike CONV-RT, raising the possibility that FLASH-RT could partly circumvent hypoxia-mediated ferroptosis resistance. However, whether this translates to durable tumor control in the hypoxic core remains unsupported [125,147].

Fractionation is an underexplored variable as well. Ferroptosis appears to contribute meaningfully to radiation-induced cell death, but its relative contribution shifts with dose per fraction: higher single-fraction doses enhance lipid peroxidation and ferroptosis, suggesting that hypofractionated regimens and stereotactic radiosurgery may engage ferroptosis more strongly than the standard 60 Gy in 30 fractions, and that pairing hypofractionation with ferroptosis inducers is a rational but untested direction [148]. Conversely, fractionation and hypoxia both reduce overall regulated cell death, and ferroptosis contribution falls under these conditions, albeit less than for necroptosis, implying an ideal but not guaranteed therapeutic niche [149]. How ferroptosis integrates with emerging delivery paradigms such as spatially fractionated RT and personalized ultrafractionated stereotactic adaptive radiotherapy (PULSAR) also represents a clear opportunity for future studies [150,151].

Ferroptosis does not act in isolation, and its crosstalk with apoptosis, autophagy, necroptosis, and pyroptosis complicates therapeutic prediction. Autophagy can drive ferroptosis yet also protects GSCs, and the balance between ferroptosis and apoptosis can be pharmacologically shifted with dual-acting compounds [27,152,153]. A deliberate under-standing of which death pathway dominates given genetic and TME contexts will be needed to avoid activating compensatory survival programs.

Selectivity remains the prevailing therapeutic challenge. Inducing ferroptosis in tumor while sparing normal brain, which is itself vulnerable to ferroptotic injury, has proven a very difficult task. Nanotechnology is the most active area addressing both selectivity and BBB penetration. Recent brain-targeting platforms, metal–organic frameworks that combine high-Z radiosensitization with antioxidant disruption, bio-mimetic and exosome-based carriers co-delivering ferroptosis inducers with TMZ, and dual-target GPX4/DHODH systems, demonstrate proof of concept for tumor-specific barrier penetration and synergy with RT and chemotherapy [39,40,106]. Whether these preclinical gains survive the heterogeneity, hypoxia, and normal-tissue constraints described above is the key translational question.

Finally, the field’s most concrete near-term need is GBM-specific clinical translation. Despite robust preclinical rationale, validated ferroptosis biomarkers and prospective trials combining ferroptosis inducers or ferroptosis-targeting nanoplatforms with radio-therapy and TMZ are lacking, and existing gene signatures remain retrospective and computationally derived [61,140,154]. Major priorities in the ongoing investigation of induced ferroptosis in GBM include biomarker-guided stratification, standardization of ferroptosis assays, rational integration with FLASH-RT, proton therapy, and PULSAR, and trial designs that preserve immune-cell function while maximizing tumor ferroptosis.

## 12. Conclusions

Glioblastoma is still one of the most difficult cancers to treat, as evidenced by the near-universal recurrence rates despite surgery plus adjuvant TMZ and RT. Ferroptosis is a rational target precisely because it attacks features that define the glioblastoma cell itself: its reliance on iron, its lipid-heavy membranes, and its high oxidative load. These are traits the tumor cannot easily discard, unlike the DNA-repair and apoptotic escape routes that so often blunt conventional therapy. Over the past several years the preclinical evidence that pushing cells toward ferroptosis improves the response to RT has grown both larger and more consistent, ranging from small-molecule inducers and repurposed drugs to brain-penetrant nanoplatforms, and it fits naturally alongside efforts to loosen the tumor’s immunosuppressive grip and combine radiation with immunotherapy. The problem is that this same vulnerability is not confined to the tumor. Normal brain tissue can be injured by ferroptosis, immune cells within the microenvironment are often the first casualties, and heterogeneity, hypoxia, and the BBB all limit any uniform strategy. What the field needs now is selectivity: killing tumor cells through ferroptosis while protecting the tissues and immune populations that an indiscriminate approach would damage. That will require reliable biomarkers, sensible pairing with newer delivery methods such as FLASH and proton therapy, and the randomized clinical trials that have yet to be done. With those pieces in place, ferroptosis might have the potential to shift the trajectory of a disease that has resisted progress for decades.

## Data Availability

No new data were created in this study. Data sharing is not applicable to this article.

## References

[1] Price M., Ballard C., Benedetti J., Neff C., Cioffi G., Waite K.A., Kruchko C., Barnholtz-Sloan J.S., Ostrom Q.T. (2024). CBTRUS Statistical Report: Primary Brain and Other Central Nervous System Tumors Diagnosed in the United States in 2017–2021. Neuro Oncol..

[2] Grochans S., Cybulska A.M., Simińska D., Korbecki J., Kojder K., Chlubek D., Baranowska-Bosiacka I. (2022). Epidemiology of Glioblastoma Multiforme-Literature Review. Cancers.

[3] Louis D.N., Perry A., Wesseling P., Brat D.J., Cree I.A., Figarella-Branger D., Hawkins C., Ng H.K., Pfister S.M., Reifenberger G. (2021). The 2021 WHO Classification of Tumors of the Central Nervous System: A summary. Neuro Oncol..

[4] Wen P.Y., Weller M., Lee E.Q., Alexander B.M., Barnholtz-Sloan J.S., Barthel F.P., Batchelor T.T., Bindra R.S., Chang S.M., Chiocca E.A. (2020). Glioblastoma in adults: A Society for Neuro-Oncology (SNO) and European Society of Neuro-Oncology (EANO) consensus review on current management and future directions. Neuro Oncol..

[5] Hartmann C., Hentschel B., Wick W., Capper D., Felsberg J., Simon M., Westphal M., Schackert G., Meyermann R., Pietsch T. (2010). Patients with IDH1 wild type anaplastic astrocytomas exhibit worse prognosis than IDH1-mutated glioblastomas, and IDH1 mutation status accounts for the unfavorable prognostic effect of higher age: Implications for classification of gliomas. Acta Neuropathol..

[6] Prajapati H.P., Ansari A. (2023). Updates in the Management of Recurrent Glioblastoma Multiforme. J. Neurol. Surg. A Cent. Eur. Neurosurg..

[7] Sung J.Y., Hwang K. (2026). Glioblastoma: Epidemiology, molecular pathogenesis, diagnosis, management, and therapeutic resistance. Mol. Biomed..

[8] Zhuo S., He G., Chen T., Li X., Liang Y., Wu W., Weng L., Feng J., Gao Z., Yang K. (2022). Emerging role of ferroptosis in glioblastoma: Therapeutic opportunities and challenges. Front. Mol. Biosci..

[9] Martínez M., Mougan I. (1998). Fatty acid composition of human brain phospholipids during normal development. J. Neurochem..

[10] Lee J.H., Lee J.E., Kahng J.Y., Kim S.H., Park J.S., Yoon S.J., Um J.Y., Kim W.K., Lee J.K., Park J. (2018). Human glioblastoma arises from subventricular zone cells with low-level driver mutations. Nature.

[11] Argenziano M.G., Neelakantan T.V., Sperring C., Adeuyan O., Higgins D.M.O., Bruce J.N., Kim B.Y.S., Dovas A., Canoll P., Lim M. (2026). Emerging roles of ferroptosis in modulating the immune landscape of glial tumours. Nat. Cell Biol..

[12] Dixon S.J., Lemberg K.M., Lamprecht M.R., Skouta R., Zaitsev E.M., Gleason C.E., Patel D.N., Bauer A.J., Cantley A.M., Yang W.S. (2012). Ferroptosis: An iron-dependent form of nonapoptotic cell death. Cell.

[13] Jiang L., Kon N., Li T., Wang S.J., Su T., Hibshoosh H., Baer R., Gu W. (2015). Ferroptosis as a p53-mediated activity during tumour suppression. Nature.

[14] Robert S.M., Buckingham S.C., Campbell S.L., Robel S., Holt K.T., Ogunrinu-Babarinde T., Warren P.P., White D.M., Reid M.A., Eschbacher J.M. (2015). SLC7A11 expression is associated with seizures and predicts poor survival in patients with malignant glioma. Sci. Transl. Med..

[15] Yang W.S., SriRamaratnam R., Welsch M.E., Shimada K., Skouta R., Viswanathan V.S., Cheah J.H., Clemons P.A., Shamji A.F., Clish C.B. (2014). Regulation of ferroptotic cancer cell death by GPX4. Cell.

[16] Yang J., Lee Y., Hwang C.S. (2023). The ubiquitin-proteasome system links NADPH metabolism to ferroptosis. Trends Cell Biol..

[17] Behera M.M., Purkait S., Ghosh A., Sable M.N., Sahu R.N., Chhabra G. (2026). Transferrin Receptor 1 Overexpression Drives Proliferation and Ferroptosis Sensitivity in Glioblastoma: A Potential Therapeutic Vulnerability. Neuropathology.

[18] Zhao H., Ji B., Chen J., Huang Q., Lu X. (2017). Gpx 4 is involved in the proliferation, migration and apoptosis of glioma cells. Pathol. Res. Pract..

[19] Miao Z., Tian W., Ye Y., Gu W., Bao Z., Xu L., Sun G., Li C., Tu Y., Chao H. (2022). Hsp90 induces Acsl4-dependent glioma ferroptosis via dephosphorylating Ser637 at Drp1. Cell Death Dis..

[20] D’Aprile S., Denaro S., Lavoro A., Candido S., Giallongo S., Torrisi F., Salvatorelli L., Lazzarino G., Amorini A.M., Lazzarino G. (2024). Glioblastoma mesenchymal subtype enhances antioxidant defence to reduce susceptibility to ferroptosis. Sci. Rep..

[21] Garcia J.H., Akins E.A., Jain S., Wolf K.J., Zhang J., Choudhary N., Lad M., Shukla P., Rios J., Seo K. (2023). Multiomic screening of invasive GBM cells reveals targetable transsulfuration pathway alterations. J. Clin. Investig..

[22] de Souza I., Monteiro L.K.S., Guedes C.B., Silva M.M., Andrade-Tomaz M., Contieri B., Latancia M.T., Mendes D., Porchia B.F.M.M., Lazarini M. (2022). High levels of NRF2 sensitize temozolomide-resistant glioblastoma cells to ferroptosis via ABCC1/MRP1 upregulation. Cell Death Dis..

[23] Xu P., Ge F.H., Li W.X., Xu Z., Wang X.L., Shen J.L., Xu A.B., Hao R.R. (2022). MicroRNA-147a Targets SLC40A1 to Induce Ferroptosis in Human Glioblastoma. Anal. Cell Pathol..

[24] Abbas S., Singh S.K., Saxena A.K., Tiwari S., Sharma L.K., Tiwari M. (2020). Role of autophagy in regulation of glioma stem cells population during therapeutic stress. J. Stem Cells Regen. Med..

[25] Buccarelli M., Marconi M., Pacioni S., De Pascalis I., D’Alessandris Q.G., Martini M., Ascione B., Malorni W., Larocca L.M., Pallini R. (2018). Inhibition of autophagy increases susceptibility of glioblastoma stem cells to temozolomide by igniting ferroptosis. Cell Death Dis..

[26] Li C., Jing J., Wang Y., Jiang H. (2024). CENPA facilitates glioma stem cell stemness and suppress ferroptosis to accelerate glioblastoma multiforme progression by promoting GBP2 transcription. Pathol. Res. Pract..

[27] Lei G., Zhang Y., Koppula P., Liu X., Zhang J., Lin S.H., Ajani J.A., Xiao Q., Liao Z., Wang H. (2020). The role of ferroptosis in ionizing radiation-induced cell death and tumor suppression. Cell Res..

[28] Huang W., He Y., Yang S., Xue X., Qin H., Sun T., Yang W. (2023). CA9 knockdown enhanced ionizing radiation-induced ferroptosis and radiosensitivity of hypoxic glioma cells. Int. J. Radiat. Biol..

[29] Kram H., Prokop G., Haller B., Gempt J., Wu Y., Schmidt-Graf F., Schlegel J., Conrad M., Liesche-Starnecker F. (2022). Glioblastoma Relapses Show Increased Markers of Vulnerability to Ferroptosis. Front. Oncol..

[30] Zhou Y., Zeng L., Cai L., Zheng W., Liu X., Xiao Y., Jin X., Bai Y., Lai M., Li H. (2025). Cellular senescence-associated gene IFI16 promotes HMOX1-dependent evasion of ferroptosis and radioresistance in glioblastoma. Nat. Commun..

[31] Dong Q., Huo J., Yan Z., Li J., Chen X., Li L., Zhen H. (2026). POT1-AS1/IGF2BP2/GPX4 promotes temozolomide resistance and radioresistance in glioblastoma by inhibiting ferroptosis. Int. J. Radiat. Biol..

[32] Hacioglu N. (2026). Integrated Transcriptomic and Single-Cell Analyses Identify HILPDA as a Hypoxia-Mediated Regulator of Ferroptotic Signaling in Glioblastoma. Int. J. Mol. Sci..

[33] Zhang X., Guo Y., Li H., Han L. (2021). FIN56, a novel ferroptosis inducer, triggers lysosomal membrane permeabilization in a TFEB-dependent manner in glioblastoma. J. Cancer.

[34] Lei G., Mao C., Yan Y., Zhuang L., Gan B. (2021). Ferroptosis, radiotherapy, and combination therapeutic strategies. Protein Cell.

[35] Wang X., Shi W., Li M., Xin Y., Jiang X. (2024). RSL3 sensitizes glioma cells to ionizing radiation by suppressing TGM2-dependent DNA damage repair and epithelial-mesenchymal transition. Redox Biol..

[36] Skeie B.S., Craig-Craven A.R., Grüner E.R., Biermann M., Riemer F., Bragstad S., Sarowar S., Behbahani M., Sleire L., Schwarzlmüller T. (2026). Phase 1 dose-escalation trial combining sulfasalazine and stereotactic radiosurgery in patients with recurrent glioblastoma. Redox Biol..

[37] Glutamate Inhibitors in Glioblastoma. ClinicalTrials.gov Identifier NCT05664464. NCT05664464.

[38] Shi L., Chen H., Chen K., Zhong C., Song C., Huang Y., Wang T., Chen L., Li C., Huang A. (2023). The DRD2 Antagonist Haloperidol Mediates Autophagy-Induced Ferroptosis to Increase Temozolomide Sensitivity by Promoting Endoplasmic Reticulum Stress in Glioblastoma. Clin. Cancer Res..

[39] Xu H., Wu Z., Tang J., Gan Y., Li J., Yu Y., Chen Y., Sui R., Liu J., Zhang Y. (2025). Ginsenoside F2-modified liposomes delivering FTY720 enhance glioblastoma targeting and antitumor activity via ferroptosis. Phytomedicine.

[40] Wang M., Lai Y., Meng H., Jin A., Zhang P., Shen Y., Lin S., Yu H., Xu W., Lehto V.P. (2026). Brain-Targeting Metal-Organic Framework Nanoplatform Reprogramming Ferroptosis Sensitivity of Glioblastoma. ACS Nano.

[41] Cao Z., Liu X., Zhang W., Zhang K., Pan L., Zhu M., Qin H., Zou C., Wang W., Zhang C. (2023). Biomimetic Macrophage Membrane-Camouflaged Nanoparticles Induce Ferroptosis by Promoting Mitochondrial Damage in Glioblastoma. ACS Nano.

[42] Liang F., Wang W., Zhuo W., Chen H., Liu X., Xu J., Wang X., Gao X. (2026). Fe(3+)-based nanovesicle mediated transferrin hijacking for glioblastoma stem cell tumoricidal treatment and postoperative recurrence inhibition. Bioact. Mater..

[43] Liu S., Sun K., Li M., Liu X., Wang P., Li M., Peng B., Wang B., Chang Y.X., Yu X.A. (2025). Quadruple synergistic amplification of ferroptosis for precision glioblastoma therapy: A luteolin-coordinated ferric ion nanoplatform. J. Nanobiotechnol..

[44] Li B., Chen X., Qiu W., Zhao R., Duan J., Zhang S., Pan Z., Zhao S., Guo Q., Qi Y. (2022). Synchronous Disintegration of Ferroptosis Defense Axis via Engineered Exosome-Conjugated Magnetic Nanoparticles for Glioblastoma Therapy. Adv. Sci..

[45] Xue K., Yang R., An Y., Ding Y., Li S., Miao F., Liu D., Chen D., Tang Q. (2024). NIR-promoted ferrous ion regeneration enhances ferroptosis for glioblastoma treatment. J. Control Release.

[46] Kashani E., Sadowski M.C., Phour J., Gheinani A.H., Hlavackova K., Haemmig S., Baumgartner U., Mueller-Wirth N., Trefny C., Abu Fakher B.S.D. (2026). Targeting the miR-19b/PPP2R5E axis enhances temozolomide response in glioblastoma via ROS-induced DNA damage. Br. J. Cancer.

[47] Matesanz-Sánchez R., Peitzsch M., Lange I., Mircetic J., Seifert M., Cordes N., Vehlow A. (2025). A novel role of exostosin glycosyltransferase 2 (EXT2) in glioblastoma cell metabolism, radiosensitivity and ferroptosis. Cell Death Differ..

[48] Song Q., Peng S., Sun Z., Heng X., Zhu X. (2021). Temozolomide Drives Ferroptosis via a DMT1-Dependent Pathway in Glioblastoma Cells. Yonsei Med. J..

[49] Sehm T., Rauh M., Wiendieck K., Buchfelder M., Eyupoglu I.Y., Savaskan N.E. (2016). Temozolomide toxicity operates in a xCT/SLC7a11 dependent manner and is fostered by ferroptosis. Oncotarget.

[50] Yang F.C., Wang C., Zhu J., Gai Q.J., Mao M., He J., Qin Y., Yao X.X., Wang Y.X., Lu H.M. (2022). Inhibitory effects of temozolomide on glioma cells is sensitized by RSL3-induced ferroptosis but negatively correlated with expression of ferritin heavy chain 1 and ferritin light chain. Lab Investig..

[51] Tong S., Hong Y., Xu Y., Sun Q., Ye L., Cai J., Ye Z., Chen Q., Tian D. (2023). TFR2 regulates ferroptosis and enhances temozolomide chemo-sensitization in gliomas. Exp. Cell Res..

[52] Sleire L., Skeie B.S., Netland I.A., Førde H.E., Dodoo E., Selheim F., Leiss L., Heggdal J.I., Pedersen P.H., Wang J. (2015). Drug repurposing: Sulfasalazine sensitizes gliomas to gamma knife radiosurgery by blocking cystine uptake through system Xc-, leading to glutathione depletion. Oncogene.

[53] Robe P.A., Martin D.H., Nguyen-Khac M.T., Artesi M., Deprez M., Albert A., Vanbelle S., Califice S., Bredel M., Bours V. (2009). Early termination of ISRCTN45828668, a phase 1/2 prospective, randomized study of sulfasalazine for the treatment of progressing malignant gliomas in adults. BMC Cancer.

[54] Banu M.A., Dovas A., Argenziano M.G., Zhao W., Sperring C.P., Cuervo Grajal H., Liu Z., Higgins D.M., Amini M., Pereira B. (2024). A cell state-specific metabolic vulnerability to GPX4-dependent ferroptosis in glioblastoma. EMBO J..

[55] Han L., Wang Z., Jiang Y., Zhou H., Zhou H. (2026). Ferroptosis and metabolic reprogramming in the immunosuppressive microenvironment of glioblastoma: Emerging mechanisms and novel strategies. Front. Immunol..

[56] Liu T., Zhu C., Chen X., Guan G., Zou C., Shen S., Wu J., Wang Y., Lin Z., Chen L. (2022). Ferroptosis, as the most enriched programmed cell death process in glioma, induces immunosuppression and immunotherapy resistance. Neuro Oncol..

[57] Hao W., Sun N., Fan Y., Chen M., Liu Q., Yang M., Yang Y., Gao C. (2024). Targeted Ferroptosis-Immunotherapy Synergy: Enhanced Antiglioma Efficacy with Hybrid Nanovesicles Comprising NK Cell-Derived Exosomes and RSL3-Loaded Liposomes. ACS Appl. Mater. Interfaces.

[58] Chen D., Yu C., Xia S., Yang S., Zhu Z., Wang G., Feng L., Guo M. (2026). Enhanced Immunotherapy for Glioblastoma Using a Cholesterol Oxidase-Loaded, Pd-Doped Cu3N Nanozyme-Based Multimodal Nanoplatform. ACS Nano.

[59] Tao L., Gong W., Yang N., Huang N., Lv F., Guo H., Meng S., Xu W., Huo M. (2026). A Mutually Reinforcing Nanoplatform Orchestrates Immunogenic Ferroptosis and Reprogramming of Tumor-Associated Macrophages for Potent Immunotherapy. Nano Lett..

[60] Zhou Q., Fu Q., Li S., Kugeluke Y., Sun Y., Sun Z. (2026). Chitosan-coated copper sulfide liposomes enhance chemo/immuno/chemodynamic therapy via enhanced ferroptosis-mediated glioma cell death. Int. J. Biol. Macromol..

[61] Wang J., Zhang G., Zheng Z., Wang Z. (2026). Ferroptosis in glioblastoma: Molecular determinants, immune crosstalk, and therapeutic opportunities. Crit. Rev. Oncol. Hematol..

[62] Wang W., Green M., Choi J.E., Gijón M., Kennedy P.D., Johnson J.K., Liao P., Lang X., Kryczek I., Sell A. (2019). CD8(+) T cells regulate tumour ferroptosis during cancer immunotherapy. Nature.

[63] Lang X., Green M.D., Wang W., Yu J., Choi J.E., Jiang L., Liao P., Zhou J., Zhang Q., Dow A. (2019). Radiotherapy and Immunotherapy Promote Tumoral Lipid Oxidation and Ferroptosis via Synergistic Repression of SLC7A11. Cancer Discov..

[64] Liao P., Wang W., Wang W., Kryczek I., Li X., Bian Y., Sell A., Wei S., Grove S., Johnson J.K. (2022). CD8(+) T cells and fatty acids orchestrate tumor ferroptosis and immunity via ACSL4. Cancer Cell.

[65] Friedmann Angeli J.P., Xavier da Silva T.N., Schilling B. (2022). CD8(+) T cells PUF(A)ing the flames of cancer ferroptotic cell death. Cancer Cell.

[66] Escuder-Rodríguez J.J., Liang D., Jiang X., Sinicrope F.A. (2024). Ferroptosis: Biology and Role in Gastrointestinal Disease. Gastroenterology.

[67] Chen C., Zhu X., Song Z., Xing Y., Jin W., Li F., Shah P.T., Song S., Geng H., Xu K. (2025). Dual-targeted bacterial outer membrane vesicles enhance glioblastoma immunotherapy by regulating tumor microenvironment and inducing IFN-γ-mediated ferroptosis. J. Nanobiotechnol..

[68] Chattopadhyay S., Hazra R., Mallick A., Gayen S., Roy S. (2024). A review on comprehending immunotherapeutic approaches inducing ferroptosis: Managing tumour immunity. Immunology.

[69] Rahaman S., Vohora D., Kamal A. (2026). Ferroptosis-induced immune modulation: A new frontier in glioblastoma therapy. Naunyn Schmiedebergs Arch. Pharmacol..

[70] Yang R., Zhu T., Jiang J., Deng M., Miao J. (2026). Ferroptosis in the tumor immune microenvironment: A double-edged sword in immunotherapy. Biochim. et Biophys. Acta (BBA) Rev. Cancer.

[71] Chen X. (2026). Immunoferroptosis: Ferroptosis meets tumor immunity and immunotherapy. Trends Immunol..

[72] Efimova I., Catanzaro E., Van der Meeren L., Turubanova V.D., Hammad H., Mishchenko T.A., Vedunova M.V., Fimognari C., Bachert C., Coppieters F. (2020). Vaccination with early ferroptotic cancer cells induces efficient antitumor immunity. J. Immunother. Cancer.

[73] Janssens S., Rennen S., Agostinis P. (2024). Decoding immunogenic cell death from a dendritic cell perspective. Immunol. Rev..

[74] Saviuk M., Turubanova V.D., De Brée S., Van Lint S., Maia T.M., Devos S., Efimova I., Braet J., Van Oudenhove L., Boons G. (2026). Ferroptosis-armed dendritic cell vaccines for glioma immunotherapy. Nat. Commun..

[75] Li L., Wang S., Duan L., Zhang L., Yan H., Chen X., Tao L., Gao Y. (2026). Ferroptosis-immune crosstalk in CNS diseases: Mechanisms and translational insights. Front. Immunol..

[76] Shi L., Liu Y., Li M., Luo Z. (2022). Emerging roles of ferroptosis in the tumor immune landscape: From danger signals to anti-tumor immunity. FEBS J..

[77] Sahu A.P., Palsa K., Shenoy G., Slagle-Webb B., Connor J.R. (2026). Tumor-associated macrophages promote ferroptosis resistance in glioblastoma by stimulating iron-loaded extracellular vesicle release. J. Neuro-Oncol..

[78] Guo F., Deng T., Yan J., Mo Y., Huang Q., Jiang Q., Mo L., Ning S., Li C. (2026). Integrative multi-omics analysis and machine learning identify M2 macrophage-induced ferroptosis resistance in glioblastoma. Funct. Integr. Genom..

[79] Zhao D., Duan L., Ren H., Zhang H., Zhang M., Liu Z., Wang H., Lu W., Shi W., Hou Q. (2026). NAMPT inhibition induces ferroptosis via mitochondrial metabolic reprogramming to enhance tumour immunogenicity in glioblastoma. Front. Immunol..

[80] Xu X., Zhang Y., Liao C., Zhou H., Wu Y., Zhang W. (2023). Impact of ferroptosis-related risk genes on macrophage M1/M2 polarization and prognosis in glioblastoma. Front. Cell Neurosci..

[81] Qian Z., Zhang Z., Bai W., Li J., Rao X., Huang G., Liu J., Wei W. (2025). Immunometabolic crosstalk between tumor- associated macrophages and ferroptotic cancer cells: Mechanisms, regulation, and therapeutic applications. Front. Immunol..

[82] Lin Z., Zou S., Wen K. (2023). The crosstalk of CD8+ T cells and ferroptosis in cancer. Front. Immunol..

[83] Tang D., Kroemer G., Kang R. (2024). Ferroptosis in immunostimulation and immunosuppression. Immunol. Rev..

[84] Kim R., Hashimoto A., Markosyan N., Tyurin V.A., Tyurina Y.Y., Kar G., Fu S., Sehgal M., Garcia-Gerique L., Kossenkov A. (2022). Ferroptosis of tumour neutrophils causes immune suppression in cancer. Nature.

[85] Dong Y., Liang T., Li J., Wu L., Gu L., Lian Y., Li J., Wang Y., Kang X., Xu Z. (2026). Ferroptosis-resistant myeloid niche shapes tumor natural evolution in glioblastoma. Cancer Lett..

[86] Liu F., Halimulati M., Luo J., Jia L., Tao Q. (2026). Friend or Foe? The Dual Role of Ferroptosis in Tumor Immunity and Immunotherapy. WIREs Mech. Dis..

[87] Wang W., Li H., Liang S., Hu Y., Ding J., Wu X., Hua D. (2025). Bridging the gap: Ferroptosis of immune cells in the tumor microenvironment. Front. Immunol..

[88] Tian J., Mao Y., Liu D., Li T., Shi L., Wang Y., Zhu C. (2025). Dual Roles of Autophagy in Radiation-Induced Brain Injury: Mechanistic Insights and Therapeutic Implications. CNS Neurosci. Ther..

[89] Lei P., Walker T., Ayton S. (2025). Neuroferroptosis in health and diseases. Nat. Rev. Neurosci..

[90] Tuo Q.Z., Bush A.I., Lei P. (2026). Ferroptosis in neurological diseases: Moving towards therapeutic intervention. Mol. Psychiatry.

[91] Benarroch E. (2023). What Is the Role of Ferroptosis in Neurodegeneration?. Neurology.

[92] Huang Q., Zhang H., Chen S., Wang Y., Zhou J. (2025). Ferroptosis in central nervous system injuries: Molecular mechanisms, diagnostic approaches, and therapeutic strategies. Front. Cell. Neurosci..

[93] Zhu R., Pan J., Min Y., Wang H., Zhang J., Liu F. (2025). Traumatic brain injury-related ferroptosis: Current perspectives. J. Mol. Med..

[94] Su J., Bian C., Zheng Z., Wang H., Meng L., Xin Y., Jiang X. (2022). Cooperation effects of radiation and ferroptosis on tumor suppression and radiation injury. Front. Cell Dev. Biol..

[95] Yang P., Li J., Zhang T., Ren Y., Zhang Q., Liu R., Li H., Hua J., Wang W.A., Wang J. (2023). Ionizing radiation-induced mitophagy promotes ferroptosis by increasing intracellular free fatty acids. Cell Death Differ..

[96] Qian X., Wang Y., Kuang P., Li B., Jiang K., Wang A., Xiong K., Hong W., Yu B., Qin G. (2026). Ionizing radiation promotes lung injury by inducing ferroptosis-driven senescence in epithelial cells via NCOA4-mediated ferritinophagy. Redox Biol..

[97] Chen L., Lin W., Le Z., Wan F., Zhang H., Geng S., Huang Q., Gao J., Hu W., Chen H. (2025). Carbon ion combined photon radiotherapy induces ferroptosis via NCOA4-mediated ferritinophagy in glioblastoma. Redox Biol..

[98] Duo K., Feng X., Tian X., Wang F., Zhao Y., Yu J., Liu Y., He Y., Cai Z. (2025). Ferroptosis inhibitors: Mechanisms of action and therapeutic potential. Cell Mol. Life Sci..

[99] Zhang X., Tian M., Li X., Zheng C., Wang A., Feng J., Hu X., Chang S., Zhang H. (2021). Hematopoietic protection and mechanisms of ferrostatin-1 on hematopoietic acute radiation syndrome of mice. Int. J. Radiat. Biol..

[100] Shi W., Wang J., Li Z., Xu S., Wang J., Zhang L., Yang H. (2024). Reprimo (RPRM) mediates neuronal ferroptosis via CREB-Nrf2/SCD1 pathways in radiation-induced brain injury. Free Radic. Biol. Med..

[101] Jiajia D., Yiping W., Enyan J., Shouwu Z., Shuai Y., Xiaojian Z., Juan J., Zhen F., Jia Z., Jikai W. (2025). Maresin-1 alleviates lipid peroxidation-induced ferroptosis after radiation-induced brain injury in mice through the RORα/NRF2 pathway. Exp. Neurol..

[102] Wei X.X., Liu H.J., Liao Z.Z., Li X.X., Ma L.L., Zou W., Zhou G.J., Tang X.Q. (2025). Repetitive transcranial magnetic stimulation alleviates radiation-induced brain injury in rats: Involving the inhibition of ferroptosis. Neurosci. Lett..

[103] Qin T., Guo L., Wang X., Zhou G., Liu L., Zhang Z., Ding G. (2024). Repetitive transcranial magnetic stimulation ameliorates cognitive deficits in mice with radiation-induced brain injury by attenuating microglial pyroptosis and promoting neurogenesis via BDNF pathway. Cell Commun. Signal..

[104] Liang J., Li L., Tian H., Wang Z., Liu G., Duan X., Guo M., Liu J., Zhang W., Nice E.C. (2023). Drug Repurposing-Based Brain-Targeting Self-Assembly Nanoplatform Using Enhanced Ferroptosis against Glioblastoma. Small.

[105] Ren G., Wang X., Li P., Wu H., Bi Z., Gao Q., Ma B., Tian Z., Liu J., Feng F. (2026). Biomimetic Nanoplatform for Targeted Glioblastoma Therapy via Concurrently Triggering GPX4/DHODH Mediated Ferroptosis. Adv. Sci..

[106] Jing L., Xiao W., Hu Z., Liu X., Yuan M. (2025). A Systematic Review of Nanoparticle-Mediated Ferroptosis in Glioma Therapy. Int. J. Nanomed..

[107] Vilaplana-Lopera N., Abu-Halawa A., Walker E., Kim J., Moon E.J. (2022). Ferroptosis, a key to unravel the enigma of the FLASH effect?. Br. J. Radiol..

[108] Huang Z., Zhou H., Huang S., Zhang S., Song S., Zhang Y., Dai Y., Weng L., Wang L., Luo Z. (2025). Amifostine–Iron Nanoparacrystalline for Tumor-Selective Immunogenic Ferroptosis. Nano Lett..

[109] Koppula P., Lei G., Zhang Y., Yan Y., Mao C., Kondiparthi L., Shi J., Liu X., Horbath A., Das M. (2022). A targetable CoQ-FSP1 axis drives ferroptosis- and radiation-resistance in KEAP1 inactive lung cancers. Nat. Commun..

[110] Emmanuel N., Li H., Chen J., Zhang Y. (2022). FSP1, a novel KEAP1/NRF2 target gene regulating ferroptosis and radioresistance in lung cancers. Oncotarget.

[111] Montay-Gruel P., Acharya M.M., Gonçalves Jorge P., Petit B., Petridis I.G., Fuchs P., Leavitt R., Petersson K., Gondré M., Ollivier J. (2021). Hypofractionated FLASH-RT as an Effective Treatment against Glioblastoma that Reduces Neurocognitive Side Effects in Mice. Clin. Cancer Res..

[112] Liljedahl E., Konradsson E., Gustafsson E., Jonsson K.F., Olofsson J.K., Ceberg C., Redebrandt H.N. (2022). Long-term anti-tumor effects following both conventional radiotherapy and FLASH in fully immunocompetent animals with glioblastoma. Sci. Rep..

[113] Böhlen T.T., Germond J.F., Petersson K., Ozsahin E.M., Herrera F.G., Bailat C., Bochud F., Bourhis J., Moeckli R., Adrian G. (2023). Effect of Conventional and Ultrahigh Dose Rate FLASH Irradiations on Preclinical Tumor Models: A Systematic Analysis. Int. J. Radiat. Oncol. Biol. Phys..

[114] Montay-Gruel P., Acharya M.M., Petersson K., Alikhani L., Yakkala C., Allen B.D., Ollivier J., Petit B., Jorge P.G., Syage A.R. (2019). Long-term neurocognitive benefits of FLASH radiotherapy driven by reduced reactive oxygen species. Proc. Natl. Acad. Sci. USA.

[115] Simmons D.A., Lartey F.M., Schüler E., Rafat M., King G., Kim A., Ko R., Semaan S., Gonzalez S., Jenkins M. (2019). Reduced cognitive deficits after FLASH irradiation of whole mouse brain are associated with less hippocampal dendritic spine loss and neuroinflammation. Radiother. Oncol..

[116] Jansen J., Kimbler A., Drayson O., Lanz B., Mosso J., Grilj V., Petit B., Franco-Perez J., Simon A., Limoli C.L. (2025). Ex vivo brain MRI to assess conventional and FLASH brain irradiation effects. Radiother. Oncol..

[117] Iturri L., Bertho A., Lamirault C., Juchaux M., Gilbert C., Espenon J., Sebrie C., Jourdain L., Pouzoulet F., Verrelle P. (2023). Proton FLASH Radiation Therapy and Immune Infiltration: Evaluation in an Orthotopic Glioma Rat Model. Int. J. Radiat. Oncol. Biol. Phys..

[118] He R., Liu Y., Li W., Xie S., Liu J., Cheng G., Zhang J. (2026). Time-dependent alterations in brain metabolites and gut microbiota following whole-brain FLASH versus conventional radiotherapy in mice. Brain Res. Bull..

[119] Hörberger F., Petersson K., Ceberg S., Bäck S., Adrian G., Ceberg C. (2025). Investigating the therapeutic potential of FLASH radiotherapy—A treatment planning study. Radiother. Oncol..

[120] Williams M.T., Regan S.L., Fritz A.L., Gollaway B.M., Mascia A.E., Vatner R.E., Perentesis J.P., Vorhees C.V. (2025). Effects of whole brain proton irradiation at conventional or ultra-high dose rate (FLASH), in adult male Sprague Dawley rats. Sci. Rep..

[121] Vozenin M.C., Montay-Gruel P., Tsoutsou P., Limoli C.L. (2026). Mechanisms, challenges and opportunities for FLASH radiotherapy in cancer. Nat. Rev. Cancer.

[122] Vilaplana-Lopera N., Kim J., Nam G., Tullis I.D.C., Paillas S., Ruan J.L., Lee P.J., Jiang Y., Park S., Hou T. (2025). Tissue-Specific Iron Levels Modulate Lipid Peroxidation and the FLASH Radiotherapy Effect. Cell Death Dis..

[123] Zhang Y., Huang C., Hu A., Wang Y., Wang W., Zhu Y., Zhou W., Qiu J., Wang J., Li J. (2026). Unraveling the Redox Mechanisms Underlying FLASH Radiotherapy: Critical Dose Thresholds and NRF2-Driven Sparing of Tissue. Int. J. Radiat. Oncol. Biol. Phys..

[124] Tang X., Wang X., Xiao K., Zhu B., Lin F., Zhou L. (2026). FLASH Radiotherapy and Organelle-Targeted Radiosensitization in Glioblastoma: A Conceptual and Translational Review. Cancers.

[125] Shibata Y., Yasui H., Higashikawa K., Miyamoto N., Kuge Y. (2019). Erastin, a ferroptosis-inducing agent, sensitized cancer cells to X-ray irradiation via glutathione starvation in vitro and in vivo. PLoS ONE.

[126] Han F., Chen S., Zhang K., Zhang K., Wang M., Wang P. (2024). Targeting Nrf2/PHKG2 axis to enhance radiosensitivity in NSCLC. NPJ Precis Oncol..

[127] Aishajiang R., Liu Z., Liang Y., Du P., Wei Y., Zhuo X., Liu S., Lei P., Wang T., Yu D. (2025). Concurrent Amplification of Ferroptosis and Immune System Activation Via Nanomedicine-Mediated Radiosensitization for Triple-Negative Breast Cancer Therapy. Adv. Sci..

[128] Zhang Z., Lu M., Chen C., Tong X., Li Y., Yang K., Lv H., Xu J., Qin L. (2021). Holo-lactoferrin: The link between ferroptosis and radiotherapy in triple-negative breast cancer. Theranostics.

[129] Guo Y., Wang H., Wang X., Chen K., Feng L. (2024). Enhancing radiotherapy in triple-negative breast cancer with hesperetin-induced ferroptosis via AURKA targeting nanocomposites. J. Nanobiotechnol..

[130] Liu J., An W., Zhao Q., Liu Z., Jiang Y., Li H., Wang D. (2024). Hyperbaric oxygen enhances X-ray induced ferroptosis in oral squamous cell carcinoma cells. Oral Dis..

[131] Yang F., Gong H., Chen S., Li J., Huang N., Wang M. (2024). Depletion of SLC7A11 Sensitizes Nasopharyngeal Carcinoma Cells to Ionizing Radiation. Protein Pept. Lett..

[132] Chen Y., Feng Y., Lin Y., Zhou X., Wang L., Zhou Y., Lin K., Cai L. (2024). GSTM3 enhances radiosensitivity of nasopharyngeal carcinoma by promoting radiation-induced ferroptosis through USP14/FASN axis and GPX4. Br. J. Cancer.

[133] Feng L., Zhao K., Sun L., Yin X., Zhang J., Liu C., Li B. (2021). SLC7A11 regulated by NRF2 modulates esophageal squamous cell carcinoma radiosensitivity by inhibiting ferroptosis. J. Transl. Med..

[134] Fang Y., Chen H., Liu Y., Jiang K., Qian Y., Wei J., Fu D., Yang H., Dai S., Jin T. (2025). NUPR1 Promotes Radioresistance in Colorectal Cancer Cells by Inhibiting Ferroptosis. J. Cell Mol. Med..

[135] Minami J.K., Morrow D., Bayley N.A., Fernandez E.G., Salinas J.J., Tse C., Zhu H., Su B., Plawat R., Jones A. (2023). CDKN2A deletion remodels lipid metabolism to prime glioblastoma for ferroptosis. Cancer Cell.

[136] Chen W., Lei C., Wang Y., Guo D., Zhang S., Wang X., Zhang Z., Wang Y., Ma W. (2023). Prognostic Prediction Model for Glioblastoma: A Ferroptosis-Related Gene Prediction Model and Independent External Validation. J. Clin. Med..

[137] Liwei Z., Hanwen L., Wenpeng Z., Weijie Y., Sifang C., Bingchang Z., Zhangyu L., Xin G., Wenhua L., Jianyao M. (2025). Robust prediction of glioma prognosis by hypoxia-induced ferroptosis genes: VEGFA-XBP1 co-expression for salvage therapy. Cancer Biol. Ther..

[138] Seerapu V.N., Amaresan R., Gopal U. (2026). From Integrated Analysis to Clinical Insight: NcRNA-Mediated Ferroptosis in Glioblastoma. Cancers.

[139] Wang T.X., Liang J.Y., Zhang C., Xiong Y., Guan K.L., Yuan H.X. (2019). The oncometabolite 2-hydroxyglutarate produced by mutant IDH1 sensitizes cells to ferroptosis. Cell Death Dis..

[140] Taki A.G., Shareef A., Arora V., Oweis R., Jyothi S.R., Singh U., Sahoo S., Chauhan A.S., Alimova F., Sameer H.N. (2026). Ferroptosis in cancer: Integrating multi-omics, AI-driven biomarkers, and translational challenges. Clin. Chim. Acta.

[141] Kang R., Liu J., Wang J., Kroemer G., Tang D. (2026). Translating ferroptosis into oncology: Challenges, opportunities and future directions. Nat. Rev. Clin. Oncol..

[142] Yeon Kim S., Tang M., Lu T., Chih S.Y., Li W. (2024). Ferroptosis in glioma therapy: Advancements in sensitizing strategies and the complex tumor-promoting roles. Brain Res..

[143] Gilbert O.E., John S., Fahim A.D., Higgins D.M. (2026). Dietary modulation of ferroptosis sensitivity in glioblastoma and other treatment-resistant cancers. Ferroptosis Oxid. Stress..

[144] Yang Z., Su W., Wei X., Qu S., Zhao D., Zhou J., Wang Y., Guan Q., Qin C., Xiang J. (2023). HIF-1α drives resistance to ferroptosis in solid tumors by promoting lactate production and activating SLC1A1. Cell Rep..

[145] Beckers C., Pruschy M., Vetrugno I. (2024). Tumor hypoxia and radiotherapy: A major driver of resistance even for novel radiotherapy modalities. Semin Cancer Biol..

[146] Das A., Bailes J.E., Barlow A., Aksenov D.P. (2026). Heterogeneity, Measurement, and Clinical Implications of Oxygenation, Cell Signaling, and Redox Biology in Glioblastoma and Adult Diffuse Gliomas, with Context from Other Brain Tumors. Antioxidants.

[147] Leavitt R.J., Almeida A., Grilj V., Montay-Gruel P., Godfroid C., Petit B., Bailat C., Limoli C.L., Vozenin M.C. (2024). Acute Hypoxia Does Not Alter Tumor Sensitivity to FLASH Radiation Therapy. Int. J. Radiat. Oncol. Biol. Phys..

[148] Wei G.X., Zhao Y.Q., Tang R., Shi H.S., Li Z.P. (2025). Ferroptosis at the intersection of radiotherapy and immunotherapy. Cancer Metastasis Rev..

[149] Kerkhove L., Geirnaert F., Coppens J., Gutiérrez A., Vandenplas H., Gevaert T., Dufait I., De Ridder M. (2026). Ferroptosis is a Pivotal Player in Radiation-induced Cell Death of Colorectal Cancer Cells. Radiat. Res..

[150] Schmitt L.G., Dohopolski M., de Vis J., Mostardeiro T.R., Mitre L.P., Youssef M., Noch E., Maher E., Sun M., Patel T. (2026). Adaptive MRI-Guided Reirradiation for High-Risk Recurrent Grade 4 Gliomas: Early Clinical Outcomes and Volumetric Response. Int. J. Radiat. Oncol. Biol. Phys..

[151] Bekker R.A., Obertopp N., Redler G., Penagaricano J., Caudell J.J., Yamoah K., Pilon-Thomas S., Moros E.G., Enderling H. (2024). Spatially fractionated GRID radiation potentiates immune-mediated tumor control. Radiat. Oncol..

[152] Xie Y., Hou T., Liu J., Zhang H., Liu X., Kang R., Tang D. (2023). Autophagy-dependent ferroptosis as a potential treatment for glioblastoma. Front. Oncol..

[153] Alkhammash A., Alotaibi G. (2026). Decoding ferroptosis and apoptosis crosstalk in glioblastoma: Molecular mechanisms, microenvironmental regulation, and therapeutic advances. Mol. Biol. Rep..

[154] Karło W., Długoń M., Gutowska I., Wszołek A., Żwierełło W. (2026). Targeting Ferroptosis in Glioblastoma: Molecular Mechanisms, Tumor Microenvironment, and Therapeutic Opportunities. Cancers.

